# Interventions currently implemented among orphans in South-Africa: a scoping review

**DOI:** 10.1080/17290376.2026.2652841

**Published:** 2026-04-10

**Authors:** Martin J. Grove, Ruan Spies

**Affiliations:** Community Psychosocial Research (COMPRES) Unit, North West University, Potchefstroom, South Africa

**Keywords:** Interventions, support, orphans, HIV/AIDS, South Africa, scoping review

## Abstract

An increase in the number of childhood orphans in South Africa is causing a greater focus of the research agenda on the implementation of interventions aimed at the psychological and developmental well-being of orphans. In South Africa, there are a variety of interventions, and they differ in their efficacy, service delivery coverage, and aims. This review aimed to provide a comprehensive account of interventions currently implemented among orphans within South Africa, which induced determining the types of OVC receiving support, the type of support provided to OVC, the geographical reach of the interventions, and the challenges and limitations hindering the effectiveness of interventions. Thereby identifying the gaps present within these aspects which should be addressed with future research endeavours. Through qualitative content analysis, this scoping review analysed 17 pieces of literature discussing intervention methods among orphans in South Africa. An analysis produced 12 categories of the types of interventions currently implemented among orphans in South Africa, namely: (1) psychological interventions, (2) educational interventions, (3) psychoeducational interventions, (4) physical resource interventions, (5) service interventions, (6) emotional interventions, (7) behavioural interventions, (8) family interventions, (9) developmental interventions, (10) recreational interventions, (11) resilience interventions, and (12) language literacy interventions. The researcher observed variations in the coverage of service delivery, methods of implementation, and target populations of interventions. Interventionists face numerous challenges when implementing interventions, such as a lack of governmental support and financial limitations. Efficacy and sustainability of these interventions remain uncertain, as does the appropriateness of addressing only specific needs of OVCs. To achieve sustainable change among OVC, reviewers recommend a blended approach where physical needs are addressed first and then psychological and developmental needs are addressed.

## Introduction

Orphanhood is a condition wherein a child has lost either or both of their parents (Ntuli, Mokgatle, & Madiba, 2020). There are approximately 147 million orphans globally, with 5,700 children obtaining orphanhood status daily (United Nations Children’s Fund [UNICEF], 2022). HIV/AIDS and poverty are the two leading factors contributing to these numbers (UNICEF, 2022). Within the South African context, these contributing factors and the effects of orphanhood on children become increasingly apparent.

Between 11.9 and 18.3 million orphans obtain orphanhood status because of HIV/AIDS, while within South Africa, approximately 8.2 million individuals are diagnosed with HIV/AIDS, making up the largest share globally (Mabaso et al., 2019; Statistics South Africa [STASSA], 2020). Furthermore, poverty plays its role in the contribution to children obtaining orphanhood status in that six out of 10 children within South Africa are multidimensionally poor; that is, they are deprived of the required resources to ensure optimal health, education, and living standards (STASSA, 2020). Poverty has contributed to the 2.6 million orphans that originate from impoverished backgrounds, with dysfunctional family dynamics, abuse, and disease being prevalent (Ntuli et al., 2020; Tomlinson, Lake, & Kleintjes, 2022). Due to factors like HIV/AIDS and poverty, most children enter an orphanage with pre-existing mental health conditions, while some develop conditions during their stay at an orphanage (Gunnar & Reid, 2019; Mackes et al., 2020). Both developmental delays and mental health disorders have long-lasting effects among orphans and can impede a child’s life in numerous ways. According to Housman (2017), children with deficits in emotional regulation tend to have lower-quality social relationships, lower academic performance, a higher rate of peer rejection, and school dropouts. Regarding deficits in behavioural regulation, children have a greater tendency to display adjustment problems, reactive aggression, lower academic performance, and decreased peer acceptance (Schwarz & Gawrilow, 2019; Whiteside, Cohen, & Strauss, 2015).

The incidence of children becoming orphans in South Africa has been steadily increasing (Mejia-Pailles, Berrington, McGrath, & Hosegood, 2020). In addition to the challenges that have already been highlighted, it is evident that orphaned and vulnerable children require interventions oriented at mitigating the effects and development of psychological disorders and developmental delays. As a result, numerous interventions to improve cognitive, psychosocial, and risk-behaviour outcomes are currently being implemented among orphans (Sitienei & Pillay, 2019; Thomas, Tan, Ahmed, & Grigorenko, 2020). Some of these interventions include community interventions aimed at education assistance, home-based care, legal protection, and psychosocial support (Schenk, 2009). Whereas others utilise approaches like (Isnaeni, Hartini, & Raymondalexas Marchira, 2021): (a) support, (b) self-help, (c) teaching-learning, (d) socialisation, and (e) task-directed approaches. Each of these different types of interventions use different techniques, such as cognitive behavioural therapy, educational support, psychological support, spiritual training, mental health interventions, and skills-based interventions (Isnaeni et al., 2021).

According to Thomas et al. (2020), the effectiveness of interventions being used among orphans is uncertain as the longitudinal efficacy of these interventions is yet to be determined. Additionally, Isnaeni et al. (2021) found that interventions oriented toward orphans worked more effectively when combined with one another. Thus, with the vast number of interventions available and the uncertainty of their effectiveness or appropriate combinations to increase efficacy, it becomes increasingly challenging to select interventions to use among orphans.

When compared to non-orphans, orphans are at a far higher risk of developing both mental disorders and developmental delays (Doku, Mensah, Ananga, & Debrah, 2019). In South Africa, orphans display high rates of depression, anxiety, and post-traumatic stress disorder when compared to non-orphans, which illustrates the need for evidence-based interventions among orphans (Cluver, Orkin, Gardner, & Boyes, 2012; Doku et al., 2019).

Frood and Purssell (2020) found numerous barriers in place that inhibit the effective execution of interventions among orphans One of these barriers is interventions that are implemented and do not meet the orphan’s required needs. According to Stover, Bollinger, Walker, and Monasch (2007), children in orphanages require various types of support that range from food and health care to education and psychosocial support. Yet, some interventionists argue that orphans only require support regarding what is required for survival, while others argue that comprehensive coverage is required (Stover et al., 2007). Regardless of coverage provided, a determining factor is the funding available, the socio-economic conditions, and the needs presented by orphans in their country of origin (Stover et al., 2007).

When taking into consideration the vast number of needs presented by orphans, the numerous interventions available, the number of variables to consider when selecting or developing an intervention, and the efficacy of these interventions being uncertain, there is a need for a comprehensive account that maps what literature states are the interventions currently in use within South Africa and the gaps present within these interventions. Considering this need, the purpose of this study was to: (a) determine the types of interventions currently implemented among orphans within South Africa, (b) determine if and what type of variations were present among interventions respective of their category, and (c) to provide a comprehensive account of the nature of the service delivery of each intervention type. With the broad research question being: *Based on the available literature, what are the current interventions being utilised among orphans in South Africa?*

## Methods

A scoping review was conducted according to the design recommendations of Arksey and O’Malley (2005) as well as the recommendations of Levac, Colquhoun, and O’Brien (2010). According to Pham et al. (2014), a scoping review is the process of systematically mapping relevant literature to a topic by identifying the key concepts, theories, and sources of evidence in the literature being reviewed. Applying this systematic process to the current study entailed (Arksey & O’Malley, 2005; Levac et al., 2010): (a) identifying the research question, (b) identifying relevant studies, (c) selecting the studies, (d) charting the data, and (e) summarising and reporting findings. The researcher (the primary reviewer) chose to involve a secondary reviewer in all aspects of the systematic mapping of literature and analysis procedure. Involving a secondary reviewer helped eliminate bias as extensively as possible.

### Identifying relevant studies

According to Arksey and O’Malley (2005), a reviewer should identify relevant literature by developing a decision plan for what the search terms will be, which databases to search, and what the inclusion/exclusion search criteria will be.

#### Decision plan of key terms

The researcher used the following key terms to produce literature within the databases: [‘Interventions’], [‘Orphans’ OR ‘Vulnerable Children’ OR ‘Orphanage’ OR ‘Children’s Home’], and [‘South Africa’ OR ‘Southern Africa’ OR ‘sub-Saharan Africa’]. The ‘AND’ Boolean operator was utilised to ensure that the literature produced from the search included a combination of all the required topics. These specific key terms were selected as they are representative of the content of the scope of enquiry required to address the research question.

#### Databases

The reviewers used the EBSCO Discovery Service via the NWU library website in addition to the PubMed and Google Scholar databases.

#### Inclusion and exclusion criteria

Literature was included for screening if it was published between 2014 and 2023 to ensure that recent literature was obtained, published in English or Afrikaans or formally translated (not by means of automated translation programs) to these languages. Full-text journal studies, peer-reviewed studies, PhD theses and masters’ dissertations, quantitative, qualitative, mixed-method studies, and mini dissertations were included. Review studies, non-peer-reviewed studies, and books or chapters from books were excluded.

### Study selection

**Figure A1.**
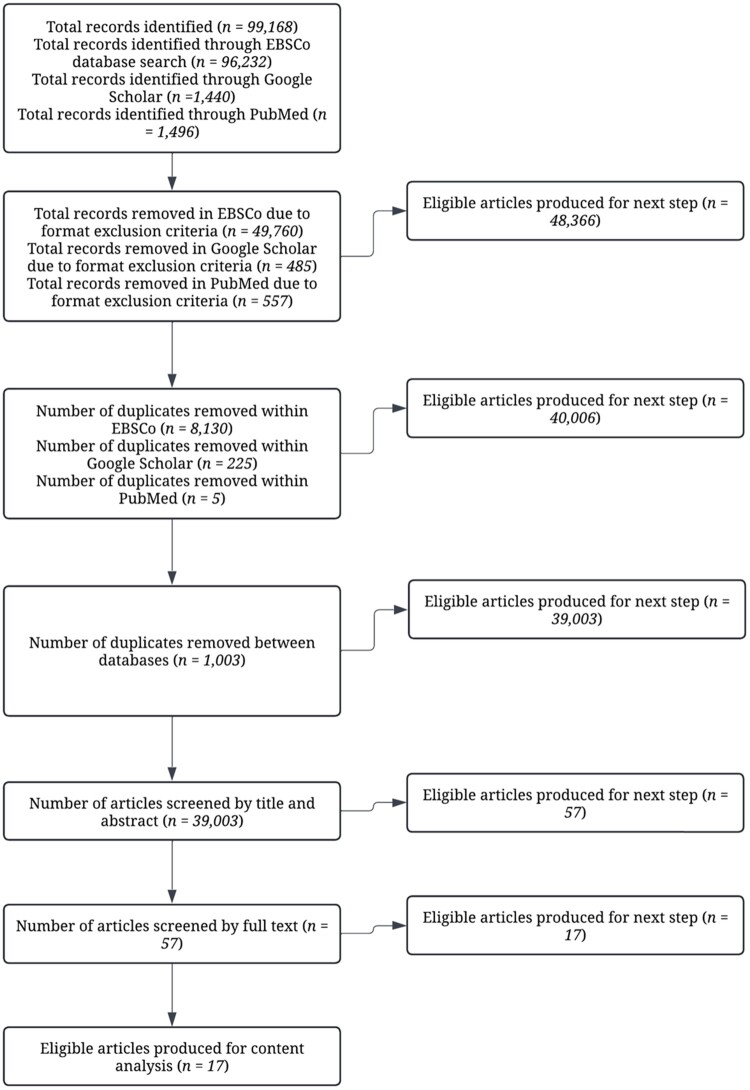
Literature selection process.

The steps in the study selection were: (1) the appropriate application of the inclusion criteria, (2) screening these results for relevant keywords within the titles and relevant information to the study contained in the abstract, and (3) full-text screening of literature which passed title and abstract screening. A secondary reviewer was involved in all three steps. Literature was only included when both the primary and secondary reviewers agreed on the relevance of the information provided. Figure A1 illustrates the selection process.

#### Charting the data

Data were extracted according to the data extraction table method as recommended by Arksey and O’Malley (2005). The primary and secondary researchers worked in conjunction to create the form. The following data points were extracted: (1) article title, (2) author(s), (3) publication date, (4) methodology, (5) participants, (6) findings or results, and (7) conclusion. Table A1 provides the information extracted from the articles selected for analysis.

### Collating, summarising, and reporting the results

The researchers synthesised the data using qualitative content analysis. According to Hsieh and Shannon (2005), qualitative content analysis is a method that helps identify themes or patterns within the content of textual data through a systemic classification process. The reviewers applied this systematic classification process by implementing five steps (Luo, 2023), namely, Step 1: select the content to be analysed; Step 2: define the meaning units and categories of analysis; Step 3: develop a set of coding rules; Step 4: code the text according to the rules, and Step 5: analyse the results and draw a conclusion.

**Figure A2.**
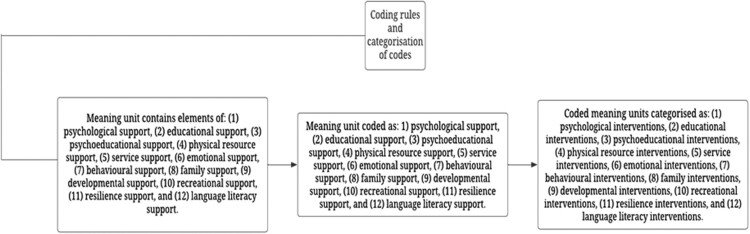
Coding rules and categorisation thereof. Note: This figure illustrates that meaning units were coded and categorised according to their respective numbers provided in the figure.

Table A2 illustrates the content that was analysed. Units of meaning pertaining to the type of intervention used in the literature was identified by the primary and secondary researcher. This allowed the researcher to induce categories by applying the coding rules. Figure A2 illustrates the coding rules and categorisation of codes. The type of support provided by each intervention informed how it would be categorised. This is in line with MacDonald et al. (2016), who stated that an intervention is any explicit attempt to provide support to an individual or a group of individuals to improve their current condition.

## Findings

Findings of the study indicated a total of 12 categories that were obtained from included literature (see Table A3). Below (see Table A2) provides the demographic information of included studies.

### Category 1: psychological interventions

The reviewers determined that psychological interventions refer to any means by which the psychological well-being of individuals is addressed (Ricou et al., 2019). This was confirmed within the context of this study from 11 sources of literature that fell under this category (Breckenridge, Black-Hughes, Rautenbach, & McKinley, 2019; Kwatubana & Ebrahim, 2020; Marais et al., 2013; Mbatha, 2014; Mokomane & Makoae, 2015; Mufalali, Makua, & Matlhaba, 2022; Mutenheri, 2014; Sitienei & Pillay, 2019; Thupayagale-Tshweneagae & Mokomane, 2014; Thurman, Kidman, Carton, & Chiroro, 2016a; Visser, Zungu, & Ndala-Magoro, 2015). The 11 relevant studies revealed a number of variations across the psychological interventions provided to OVC.

#### Variations in types of OVC supported

Five of these studies focused on children with HIV/AIDS status, one on homeless children, and one on children from child-headed homes. The remaining four studies did not target specific groups.

#### Variation in geographical reach of service delivery

Variations in service provision coverage were observed. Analysis revealed that only three out of 11 studies provided psychological interventions to OVC across multiple provinces, with the remaining nine only providing interventions within specific provinces, with five providing wide-spread interventions.

#### Variation in type of support provided

Further variations were also found with regard to the aims of the psychological interventions and how they were implemented. An NGO in the Eastern Cape provided psychological support to HIV/AIDS orphaned children through cognitive behavioural therapy, leading to a decline in destructive or low self-esteem behaviours (Breckenridge et al., 2019). A community-based organisation in Soweto provided psychological support to OVC through mentorship and peer-group programmes, fostering strong social ties and providing guidance (Sitienei & Pillay, 2019). The South Africa Red Cross Society (SARCS) provided psychological support to HIV/AIDS-affected OVC in Matatiele Local Municipality, improving their well-being through health education, indoor and outdoor activities, and gardening skills development (Mufalali et al., 2022). This support helped OVC develop positive coping mechanisms, improve school attendance and academic performance, and maintain healthy relationships, ultimately leading to improved resilience and socialisation (Mufalali et al., 2022).

Although the three psychological interventions mentioned in the previous paragraph were successful in improving the psychological well-being of OVC, these interventions were isolated to specific areas and provided support to a limit number of children. In contrast to the limited service delivery area and number of children receiving these services, health programme coordinators from five schools in Gauteng's Sedibeng East district reported psychological interventions that provided support to CHH learners through social workers, Lifeline counsellors and pastors, as well as counselling sessions on HIV/AIDS and teenage pregnancy (Kwatubana & Ebrahim, 2020).

In the Mangaung Municipality area of the Free State, five CBOs provided psychological support to OVC (Marais et al., 2013): (a) improving community and family capacities to provide psychological support to OVC, (b) improving referral mechanisms for OVC to access specialised psychological services, and (c) providing on-going support programs to OVC. Three schools in Ntuzuma G-section KwaZulu-Natal offered psychological support to OVC students through school-based programmes, with teachers acting as lay counsellors, addressing their basic psychological needs (Mbatha, 2014).

The James House Isibindi programme in Cape Town offered psychological support to OVC through grief counselling, memory boxes, HIV/AIDS testing counselling, and specialised therapy (Mutenheri, 2014). The Future Families programme in Pretoria offered psychological support to OVC and their families in three peri-urban townships, focusing on HIV/AIDS counselling and stigmatisation (Thurman et al., 2016a).

Although the abovementioned psychological interventions were provided to OVC in several areas, these interventions only had selected service areas within South Africa, respective to the province wherein they function. One programme implemented widely across South Africa is the *Better Accept Reality* (BAR) programme, which offered psychological support to OVC through training and education to foster open communication, assertiveness, and problem-solving skills (Thupayagale-Tshweneagae & Mokomane, 2014). While street shelters for homeless children within four different provinces provided psychological support by means of focussing on early intervention and therapeutic programs (Mokomane & Makoae, 2015). Furthermore, the ISIBINDI programme in KwaZulu-Natal, Eastern Cape, Mpumalanga, and Gautengwas providing psychological support to OVC through personal guidance and counselling to enhance self-esteem, problem-solving, and interpersonal skills (Visser et al., 2015).

### Category 2: educational interventions

The reviewers had determined that educational interventions refer to any means by which the educational experience and/or abilities of the individual is being improved (Berninger, Fayol, & Alston-Abel, 2011). This was confirmed within the context of this study from the seven sources of literature included in this category (*n* *=* *7*; Balie & Sayed, 2020; Eale, 2018; Mbatha, 2014; Mutenheri, 2014; Mwoma & Pillay, 2015; Sitienei & Pillay, 2019; Visser et al., 2015). The eight studies displayed numerous variations across the educational interventions provided to OVC.

#### Variations in types of OVC supported

Eight studies indicated that education support was provided to OVC, with three of the featured programmes focusing on HIV/AIDS-affected OVC within their service areas, one on CHHs, and four on children who retained OVC status.

#### Variation in geographical reach of service delivery

It was noted that only one study provided educational support to OVC in multiple provinces, while seven studies only provided support in specific areas, with two indicating widespread support within these areas.

#### Variation in type of support provided

Further variations were also found with regard to the aims of the educational interventions and how they were implemented. A primary school in Soweto received support from governmental and non-governmental sources for the educational needs of OVC. Governmental sources provided meals, school fees, books, and stationery, while non-governmental organisations provided uniforms. Teachers provided motivational support and aided in the completion of schoolwork for OVC (Mwoma & Pillay, 2015).

Similarly, one CBO in Soweto provided education support to OVC by providing school uniforms, educational materials, and food hampers, as well as paying for tuition and school-related excursions (Sitienei & Pillay, 2019).

The Future Families programme in Gauteng offers was offering educational support to OVC by providing: (1) a safe school environment, (2) motivational talks, (3) homework support, (4) after-school tutorials, (5) support for learning difficulties, (6) referrals to schools, (7) funding for school uniforms, and (8) school attendance and work completion monitoring (Eale, 2018).

Although it is essential that the tangible educational needs of OVC be addressed, another important factor to consider is the educational culture that teachers implement among OVC (Balie & Sayed, 2020). In the Western Cape, one child and youth care centre (CYCC) implemented the Curriculum of Care, focusing on the educational needs of at-risk children and youth. This curriculum involves teachers building relationships with OVC, identifying and addressing their needs, and addressing emotional and psychological barriers to ensure inclusion in learning (Balie & Sayed, 2020).

Although the above-mentioned educational interventions were effective in providing educational support to OVC, these interventions only provided support to a limited number of OVC because their respective service areas had a limited geographical reach. In contrast to this limited scope of service delivery, three schools in the Ntuzuma G-section in KwaZulu-Natal provided educational support to OVC students, focusing on addressing the basic needs of OVC. Teachers focussed on the psychological and emotional well-being of OVC to motivate and support them. OVC students also received food from the schools’ nutritional programmes and school uniforms through donations (Mbatha, 2014). Within the Hout Bay area of Cape Town, the James House Isibindi programme addressed the educational needs of OVC in five ways (Mutenheri, 2014). First, by aiding OVC with school registration, and second, by regularly following up with schools to determine their progression and if problems are present which require further educational support. The third form of support was providing homework assistance; fourth, improving access to resources such as libraries and the internet, and lastly, by providing school uniforms, stationary, educational resources, or payment of transportation to and from schools. We see that these educational interventions provided wide-spread educational support to OVC, yet the support was limited to the provinces wherein the interventions’ service delivery functioned.

In contrast to this, the ISIBINDI programme offers more wide-spread comprehensive educational support to OVC as the programme’s support stretches through KwaZulu-Natal, the Eastern Cape, Mpumalanga, and Gauteng, covering schoolwork, tuition, further education, training, bursary applications, job skills, and career guidance (Visser et al., 2015).

### Category 3: physical resource interventions

The reviewers had determined that physical resource interventions refer to any attempt/s made to improve the quality of life by providing tangible support in the form of physical resources (Bradley, Lloyd-Williams, & Dowrick, 2018). This was confirmed within the context of this study from the five sources of literature included in this category (Eale, 2018; Marais et al., 2013; Mutenheri, 2014; Thurman, Luckett, Taylor, & Carnay, 2016b; Visser et al., 2015). Variations in type of OVC supported, scope of service delivery coverage, and types of support provided were observed within the studies.

#### Variation in type of OVC supported

Of the five studies included, two indicated that support was only provided to OVC affected by HIV/AIDS. While the remaining three studies indicated that support was provided to children who retained OVC status.

#### Variation in geographical reach of service delivery

Further variations were also observed with regard to service delivery areas, with one study indicating that physical resource support was widespread while the remaining four indicated that physical resource support was restricted to their respective provinces.

#### Variation in type of support provided

Lastly, variations were also observed with regard to the means by which physical resource support was provided. The Future Families home visit programme provided support to OVC in Pretoria with regards to HIV testing, while the Future Families programme in Olievenhoutbosch provided support to OVC through gardening support to ensure that OVC has their nutritional needs met (Eale, 2018; Thurman et al., 2016b). Both intervention efforts were limited in terms of their service delivery area and could, therefore, only provide physical resource support to a limited number of OVC. In contrast, five CBOs within the Free State area provided support to OVC through food packages, nutritional supplements, and care facilities such as children's homes, temporary shelters, and daycare facilities (Marais et al., 2013). The James House Isibindi programme provided OVC with a safe park in which to play to reduce exploitation and abuse among OVC (Mutenheri, 2014).

Although some interventions provided broader support in terms of service area, physical resource interventions were still limited to OVC within their respective provinces. Whereas the Isibindi programme provided OVC with food gardens and safe parks to play in within the broader reach of KwaZulu-Natal, Eastern Cape, Mpumalanga, and Gauteng (Visser et al., 2015).

### Category 4: psychoeducational interventions

The reviewers had determined that psychoeducational interventions refer to any means by which education and other supportive interventions are combined to improve knowledge and functioning (Morgado, Lopes, Carvalho, & Santos, 2022). This was confirmed within the context of this study from the six sources of literature included in this category (Kwatubana & Ebrahim, 2020; Marais et al., 2013; Sitienei & Pillay, 2019; Thupayagale-Tshweneagae & Mokomane, 2014; Thurman et al., 2016a; Visser et al., 2015). Variations in types of OVC supported, geographical reach of service delivery, and type of psychoeducational support provided were observed.

#### Variation in type of OVC supported

Four of the six studies included in this category indicated that psychoeducational support was oriented towards OVC who have been affected by HIV/AIDS. In contrast, one study indicated that psychoeducational support was provided to children retaining OVC status.

#### Variation in geographical reach of service delivery

Four studies indicated that service delivery only occurred within their respective provinces, while one study indicated that psychoeducational support was provided widely across South Africa.

#### Variation in type of support provided

Further variations were also observed with regard to the aims of the psychoeducational interventions provided in addition to the means by which support was provided. Within Soweto, one CBO offered life skills training focusing on teaching OVC how to respect and interact with one another, what HIV/AIDS is and how to protect themselves, how to use condoms, and how to avoid sex that leads to teenage pregnancy (Sitienei & Pillay, 2019). Similarly, five schools in Gauteng's Sedibeng East district provide psychoeducational interventions to CHH students, with Lifeline nurses and counsellors regularly discussing health issues like teenage pregnancy and AIDS while addressing other health concerns of students (Kwatubana & Ebrahim, 2020).

The Vhutshilo intervention in two Eastern Cape rural districts provides psychoeducational support to OVC, addressing HIV risk factors, alcohol and substance abuse, crime, sexual violence, and condom use (Thurman et al., 2016a). Additionally, the five CBOs within the Free State who cater towards OVC provided skill training and education with regard to HIV/AIDS by means of support groups (Marais et al., 2013).

Although these interventions were effective in addressing the psychoeducational needs of OVC, service delivery was limited to their respective provinces. In contrast to this, the ISIBINDI project provided life skills training with regards to HIV, the risks thereof and how to not contract the disease in addition to substance abuse education to OVC in KwaZulu-Natal, the Eastern Cape, Mpumalanga, and Gauteng (Visser et al., 2015). The BAR programme offered psychoeducational support to OVC widely across South Africa, focusing on educating OVC on aspects such as death and dying, implementing appropriate behaviours, problem-solving skills, and asserting needs (Thupayagale-Tshweneagae & Mokomane, 2014).

### Category 5: emotional interventions

The reviewers had determined that emotional interventions refer to any means by which the emotional well-being of the individual is being addressed in such a way that the management and experience thereof is improved (Hassani & Schwab, 2021). This was confirmed within the context of this study from the five sources of literature included in this category (*n* *=* *5*; Balie & Sayed, 2020; Breckenridge et al., 2019; Mbatha, 2014; Thurman et al., 2016a, 2016b). Variations in types of OVC supported, geographical reach of service delivery, and type of emotional support provided were observed.

#### Variation in type of OVC supported

Of the five studies included, one indicated that emotional support was only provided to OVC affected by HIV/AIDS. In contrast, another indicated that service delivery had only occurred to children from CHH. The remaining three studies indicated that emotional support was provided to children who retained OVC status.

#### Variation in geographical reach of service delivery

With regard to the area of service delivery, four of the studies indicated that their service delivery was limited to certain provinces and or areas within their respective provinces. While one study indicated widespread service delivery across South Africa.

#### Variation in type of support provided

Further variations were also observed with regard to how emotional interventions were provided to OVC. In Gauteng’s Sedibeng East district, children from CHHs receive emotional support from social workers and pastors, focusing on emotional wellness and discussing experiences of being OVC with one another during sessions (Kwatubana & Ebrahim, 2020). The Networks of Hope programme provided interpersonal psychotherapy to OVC in rural districts of the Eastern Cape, facilitating dialogue between OVC to share their problems and emotions experienced to help one another (Thurman et al., 2016a, 2016b).

In the Eastern Cape, an NGO in a rural village provided emotional support to orphans affected by HIV/AIDS using cognitive behavioural and psychoeducational strategies, leading to increased positive emotions and emotional expression (Breckenridge et al., 2019). Teachers within the Ntuzuma G-section of KwaZulu-Natal received in-service training in order to assist OVC with their emotional needs by applying basic counselling skills in the school (Mbatha, 2014).

Although the above-mentioned interventions were effective in addressing the emotional needs of OVC, their services were limited to either a specific province or within particular areas within their respective provinces. In contrast, the Curriculum of Care is implemented widely across South Africa among OVC within schools. The curriculum focusses on the creation of a culture of care among teachers and OVC to develop caring relationships with OVC (Balie & Sayed, 2020). Teachers then use the relationship to gain an understanding of and address the emotional needs of OVC (Balie & Sayed, 2020).

### Category 6: service interventions

The reviewers had determined that service interventions refer to any means by which the individual’s access to public, social and or governmental services have been improved. This was confirmed within the context of this study from the five sources of literature included in this category (*n* *=* *5*; Eale, 2018; Marais et al., 2013; Mutenheri, 2014; Thurman et al., 2016b; Visser et al., 2015). Variations in types of OVC supported, geographical reach of service delivery, and type of service support provided were observed.

#### Variation in type of OVC supported

Of the five studies included in this category, one indicated that service interventions were provided to OVC affected by HIV/AIDS. In comparison, the remaining four studies indicated that service delivery was provided to children retaining OVC status.

#### Variation in geographical reach of service delivery

Four studies indicated that service interventions were only provided to OVC within certain provinces and or specific areas within their respective provinces. In comparison, one study indicated that service interventions were provided to OVC widely across South Africa.

#### Variation in type of support provided

Variations were also observed in how service interventions were provided to OVC. CBOs in the Free State aided OVC and their caregivers by improving access to social grants, birth certificates, food parcels, and nutritional supplements (Marais et al., 2013). The James House programme offered referrals for healthcare services and governmental grants, while the Future Families programmes provided referrals to health and social services to promote the update of HIV testing and treatment (Eale, 2018; Mutenheri, 2014; Thurman et al., 2016b).

The service interventions discussed above were successful in their attempts to provide information to OVC and caregivers and in improving access to services, however, these interventions were only provided to OVC and caregivers within certain provinces and or areas within their respective provinces. In contrast to this, the ISIBINDI programme facilitated inter-sectoral collaboration and referrals to OVC and caregivers in KwaZulu-Natal, Eastern Cape, Mpumalanga, and Gauteng, improving access to educational, healthcare, social services, and child grants (Visser et al., 2015).

### Category 7: behavioural interventions

The reviewers had determined that emotional interventions refer to any means by which attempts are made to alter an individual’s behaviour to improve their quality of life (Yeung et al., 2015). This was confirmed within the context of this study from the three sources of literature included in this category (*n* *=* *3*; Balie & Sayed, 2020; Breckenridge et al., 2019; Marais et al., 2013). Variations in types of OVC supported and type of behavioural support provided were observed. All three studies indicated that service delivery was restricted to their respective provinces and/or specific areas within their provinces.

#### Variation in type of OVC supported

Of the three studies included in this category, only one indicated that behavioural interventions were oriented towards OVC affected by HIV/AIDS, whereas the remaining two indicated that behavioural interventions were oriented towards children retaining OVC status.

#### Variation in type of support provided

Five CBOs in Mangaung Municipality, Free State, offered behavioural modification programmes through workshops, door-to-door campaigns, exhibitions, public meetings, and educational materials to educate OVC on appropriate behaviours (Marais et al., 2013). While one NGO in the Eastern Cape provided behavioural interventions by employing behavioural and psychoeducational strategies to improve the interpersonal abilities of OVC (Breckenridge et al., 2019). Additionally, the Curriculum of Care enabled teachers to have a better approach to addressing the behavioural barriers that OVC face in educational settings (Balie & Sayed, 2020). By forming a caring relationship with the students, teachers could employ lay counselling methods to identify and address the behavioural barriers faced by OVC (Balie & Sayed, 2020).

### Category 8: developmental interventions

The reviewers had determined that developmental interventions refer to any means by which attempts are made to facilitate normal developmental milestone occurrences (Belcher & Palenberg, 2018). This was confirmed within the context of this study from the source of literature included in this category (*n* *=* *1*; Mokomane & Makoae, 2015). No variations were observed due to only one study indicating developmental interventions were provided to OVC.

The one study included in this category indicated that developmental interventions were provided to street children and were widely implemented across South Africa. Shelters within four provinces in South Africa offered a programme focusing on the developmental needs of street children. These shelters fostered healthy family dynamics and healthy relationships between parents and children. They also educated children on hygiene and nutrition and enrolled them into schools. These measures aimed to provide the necessary environment, knowledge, and exposure for normal development (Mokomane & Makoae, 2015).

### Category 9: recreational interventions

The reviewers had determined that recreational interventions refer to any means by which recreational activities are employed to improve the well-being of the individual. This was confirmed within the context of this study from the source of literature included in this study (*n* *=* *1*; Mokomane & Makoae, 2015). No variations were observed due to only one study indicating that recreational support was provided to OVC.

The one study included in this category indicated that developmental interventions were provided to street children and were widely implemented across South Africa. A 2014 audit of South African street children's shelters revealed recreational programmes as a form of intervention. These recreational programmes included indoor and outdoor activities like soccer, board games, television, and arts and crafts (Mokomane & Makoae, 2015).

### Category 10: resilience interventions

The reviewers had determined that resilience interventions refer to means by which the individual’s quality of life is improved by restoring normal functioning and improving adaptability and adjustment (Wang, Chi, Zhan, Chen, & Li, 2021). This was confirmed within the context of the study from the source of literature included in this category (*n* *=* *1*; Braband, Faris, & Wilson-Anderson, 2018). No variations were observed due to only one study indicating that resilience support was provided to OVC.

The one study included in this category indicated that the intervention was not only widely implemented across South Africa but was also implemented in India and Kenya. The intervention was oriented towards children within institutionalised care because of their OVC status. The Memory Book intervention enhanced resilience among OVC by encouraging OVC to engage in drawing and storytelling activities and incorporating mementoes of their parents, thereby facilitating non-threatening trauma processing (Braband et al., 2018).

### Category 11: family interventions

The reviewers had determined that family interventions refer to any means by which the needs of family members or caregivers of the individual are addressed or by involving family members or caregivers in an individual’s treatment (Varghese, Kirpekar, & Loganathan, 2020). This was confirmed by the three sources of literature included in this category (Eale, 2018; Sitienei & Pillay, 2019; Visser et al., 2015). Only variations in type of support provided were observed.

Of the three included studies, all indicated that family interventions were provided to OVC and family members within their respective provinces or specific areas within those provinces. All these studies indicated that family interventions were provided to children retaining OVC status.

#### Variation in type of support provided

The CBO in Soweto helped OVC families with house rental payments and food supplies (Sitienei & Pillay, 2019). The ISIBINDI project and Future Families programmes in Olievenhoutbosch also supported families through home visits, promoting open communication and involving family members in a variety of services (Eale, 2018; Visser et al., 2015).

### Category 12: language and literacy interventions

The reviewers had determined that language and literacy interventions refer to any means by which the individual’s language and literacy abilities are improved (Oxley & de Cat, 2019). This was confirmed by the one source of literature included in this category (*n* *=* *1*; Zoetmulder, 2019). No variations were observed as only one study was included in this category.

The study included in this category indicated that language and literacy interventions were provided to children who retained OVC status within one specific province. The Durban Child Youth Care Centre (DYCC) in Glenwood, Kwazulu-Natal, offered language-literacy support intervention for OVC from grades 1–3 and involved volunteers and language and speech therapists (Zoetmulder, 2019).

## Discussion

The literature review examined 17 articles, all of which discussed interventions employed among orphans in South Africa. The primary findings of this study indicate that, at the time of this review, there were 12 types of interventions employed among orphans in South Africa. These types are listed as follows in order from most to least implemented: (1) psychological interventions, (2) educational interventions, (3) psychoeducational interventions, (4) physical resource interventions, (5) service interventions, (6) emotional interventions, (7) behavioural interventions, (8) family interventions, (9) developmental interventions, (10) recreational interventions, (11) resilience interventions, and (12) language literacy interventions.

### Psychological interventions

OVC in South Africa are at a high risk for numerous negative outcomes, especially pertaining to their psychological well-being (Allman et al., 2022). This appears to be a widespread shared sentiment since psychological interventions were implemented in 11 of the 17 included studies.

#### Geographical spread of interventions

Psychological interventions were provided to OVC in the Eastern Cape, KwaZulu-Natal, Gauteng, and the Free-State. Although this may indicate that service provision of psychological interventions is widespread within South Africa, this still only accounted for 63.9% of orphans in South Africa (Hall, 2019). Furthermore, some services were restricted to only certain areas within the provinces and only provided services to certain types of OVC, thereby decreasing the percentage of OVC obtaining psychological support even further. Given the high prevalence rate of psychological disorders among OVC in South Africa, this is concerning (Allman et al., 2022).

#### Types of psychological interventions provided

Psychological interventions were provided in the forms of: (a) promoting positive coping mechanisms to improve school attendance and performance, prosocial behaviours, and interpersonal relationships (Mufalali et al., 2022), (b) mentorship and peer-group support (Sitienei & Pillay, 2019), (c) personal guidance and counselling (Visser et al., 2015), (d) counselling of traumatic experiences and related symptomology (Kwatubana & Ebrahim, 2020), (e) enhancing community and family capacity to care for OVC with psychological disorders and referral mechanisms (Marais et al., 2013), (f) cognitive behavioural therapy (CBT) to support OVC dealing with domestic violence, community safety, bullying, and schoolwork completion (Breckenridge et al., 2019), (g) lay counselling and referrals (Mbatha, 2014), (h) counselling to OVC and their families about HIV, the testing thereof, and the associated stigma (Thurman et al., 2016a), (i) peer-based training and education to enhance communication and problem-solving skills among OVC (Thupayagale-Tshweneagae & Mokomane, 2014), (j) therapeutic programs which focused on the basic needs of OVC, developmental assessment, and obtaining collateral information (Mokomane & Makoae, 2015), and (k) grief counselling, memory boxes, HIV/AIDS testing, and specialised therapy (Mutenheri, 2014).

Despite the variety of psychological interventions provided to OVC, there are numerous challenges and limitations that impede their effectiveness.

#### Challenges and limitations of interventions

The promotion of positive coping mechanisms may be effective in improving school attendance and performance as well as being beneficial to prosocial behaviours and interpersonal relationships; however, the challenge lies in fostering awareness among families and communities about the psychological issues among OVC. Involving both family and community members in implementing psychological interventions is crucial for the success of such interventions (Mufalali et al., 2022). Mentorships are a practical and direct way to provide individual psychological support and can improve psychological well-being. In contrast, peer-group support moderately improves overall well-being but doesn't address individual psychological aspects like hope or empowerment (Lyons, Cooper, & Lloyd-Evans, 2021). Therefore, it is important to consider the specific psychological aspect that needs to be addressed when selecting and applying a psychological intervention, for example, when choosing between a mentorship or peer-group intervention (Beutler et al., 2016).

OVC often face challenges like poverty, abuse, neglect, and loss of parents, leading to lower self-reliance and identity development (Zhang et al., 2022). According to Mathwasa and Sibanda (2020), both personal guidance and counselling interventions can enhance self-esteem, problem-solving skills, and resilience. This was confirmed in the literature, which showed that participants experienced improved self-reflection, insight, and commitment to self-improvement (Visser et al., 2015). Therefore, personal guidance as a psychological intervention proved to be effective in improving the psychological well-being of OVC (Visser et al., 2015).

Zhang et al. (2022) suggested that counselling can significantly improve the psychological well-being of OVC by enhancing social support and post-stress growth, thereby addressing the challenges faced by OVC by providing social support and improving post-stress growth. Lay counselling that focusses on traumatic experiences and related symptomology has shown inconsistent results in reducing trauma-related symptoms (Han et al., 2021). However, lay counselling as a type of psychological intervention is effective in improving trauma-related symptomology, ensuring normal development for OVC (Connolly et al., 2021). As trauma lies at the core of OVC experiences, it is crucial to choose the appropriate means of treatment in order to ensure that OVC undergo normal development (Gregorowski & Seedat, 2013)

Interventions that address the barriers to social support faced by OVC and caregivers are crucial for improving psychological well-being because they increase social support by enhancing community and family capacity and referral mechanisms (Zhang et al., 2022). However, CBOs face financial constraints, limiting their capacity to provide this type of psychological support (Marais et al., 2013). The BAR programme focussed on peer-based training and education to enhance communication and problem-solving skills among OVC, which significantly improves resilience while reducing anxiety and depression, which are crucial for effective problem-solving and resilience (Thupayagale-Tshweneagae & Mokomane, 2014).

CBT has been shown to improve psychological well-being and self-confidence, preventing destructive behaviours (Halder & Mahato, 2019). However, it should be combined with psychoeducational approaches to educate the family or caregivers of the child or adolescent (Halder & Mahato, 2019). Furthermore, the effectiveness of therapeutic programs varies depending on the background of the OVC and must be considered (Thomas et al., 2020). Addressing the basic needs of OVC, such as food and shelter, should be addressed first (Thomas et al., 2020). Schools play a crucial role in the psychological well-being of OVC as they predominantly rely on the school and its environment for support. Still, limited resources in rural areas limit the support that can be provided and necessitate involving family and community members for comprehensive coverage (Thabethe, Mbatha, & Mtapuri, 2016). A societal-community-school system is needed to ensure comprehensive coverage for OVC (Thabethe et al., 2016). While HIV education through counselling is crucial in the process of ensuring HIV treatment uptake improves among OVC. Which can lead to earlier diagnoses, more effective treatment, and reduced mortality (Thurman et al., 2016a, 2016b).

### Educational interventions

Educational interventions were the second-most common intervention implemented among OVC and were implemented in eight of the 17 articles. The educational interventions varied in terms of the focus and methods. According to Pillay (2018), education is crucially important for OVC as it can significantly improve their quality of life by conferring knowledge and skills to them. Furthermore, schooling can contribute to the social integration of OVC and provide them with a safe and structured environment (Mwoma & Pillay, 2015). As a result, ensuring that OVC are able to function properly in school is of crucial importance to their academic and psychological well-being (Pillay, 2018).

### Geographical Spread of Interventions

Educational interventions were provided to OVC in KwaZulu-Natal, the Eastern Cape, Mpumalanga, Gauteng, and the Western Cape. These five provinces account for 70.9% of the OVC population (Hall, 2019). However, the percentage of reached OVC decreases when taking into consideration that service delivery was restricted to only certain areas in the provinces, that service delivery was provided only to certain types of OVC in some cases, and that intervention efforts faced numerous barriers and limitations. These limitations pose a concern with regard to the effectiveness of these interventions when considering that the majority of orphans struggle to successfully obtain access to or complete their education (Pillay, 2018).

#### Types of educational interventions provided

In the scope of this review, educational interventions were provided in the forms of: (a) providing educational support, including schoolwork assistance, tuition assistance, and career guidance (Visser et al., 2015), (b) paying tuition fees, providing books, food, uniforms, and academic assistance (Mwoma & Pillay, 2015), (c) addressing the unique educational needs of OVC and focused on building positive relationships between teachers and OVC (Balie & Sayed, 2020), (d) providing aid with regards to basic needs of OVC, such as school fees, uniforms, and food (Mbatha, 2014), (e) providing motivational and material support to create a safe environment, improving OVC’s school attendance and academic performance (Eale, 2018), (f) provision of material resources to OVC and caregivers required for the education of OVC (Mutenheri, 2014), (g) providing uniforms and food hampers to create a safe environment (Kwatubana & Ebrahim, 2020), and (h) providing educational support through the provision of educational materials and rewarding students for good academic performance, and paying fees (Sitienei & Pillay, 2019).

Despite the variety of educational interventions provided to OVC, there are numerous challenges and limitations that impede their effectiveness.

#### Challenges and limitations of interventions

OVC often face poorer educational outcomes, higher dropout rates, and lower university attendance due to poverty (Pillay, 2018). Therefore, the provision of educational support focussing on schoolwork assistance, tuition assistance, and career guidance is crucial (Pillay, 2018). Even if education support is successful, high school completion doesn’t guarantee employment for most OVC, and the lack of child grants provided to caregivers once OVC turn 18 results in extra financial stress. Financial stress may cause the OVC to resort to crime or transactional sex to obtain an income (Visser et al., 2015). In addition, a lack of quality education can hinder employment opportunities, income generation, and general well-being, especially for OVC (Mwoma & Pillay, 2015). Economic hardships and exposure to abuse, neglect, and exploitation further complicate the situation (Ntuli et al., 2020). In South Africa, sustainable education is crucial and requires involvement from various stakeholders because the deteriorating community network has placed more responsibility on external role players, including governmental and non-governmental organisations (Ntuli et al., 2020).

Challenges such as limited resources available to the school, students being unable to concentrate, and service provision only being provided to OVC within the school resulted in limited effectiveness of providing OVC with books, food, school uniforms, schoolwork assistance and payment of tuition fees (Mwoma & Pillay, 2015). School absenteeism was prevalent among OVC, which limited the number of OVC that received support through these interventions. Those who did attend school only had access to support that was not longitudinally sustainable (Mwoma & Pillay, 2015). The use of Curriculum of Care focussed on making curriculum changes in order to build positive relationships between teachers and OVC (Balie & Sayed, 2020). The challenge is that students were expected to participate in a full schedule, often at the expense of a deep learning experience. The solution could be to take a more balanced approach to the curriculum to address the educational and psychological needs of OVC (Balie & Sayed, 2020)

Meeting the basic needs of children is crucial for their optimal development. As such, helping OVC obtain access to uniforms and food and to pay tuition fees is crucial (Mbatha, 2014). However, government assistance was insufficient, with schools often relying on donations that were not sustainable. A collaborative effort between schools, the Department of Education, and communities is necessary (Mbatha, 2014).

The school environment is crucial for OVC students to feel safe and secure. A positive environment can prevent feelings of isolation and low self-esteem, as well as improve attendance and academic performance (Edgerton & McKechnie, 2023). Therefore, addressing the motivational and material needs of OVC is crucial to allow for a safe school environment for OVC (Eale, 2018). By addressing both the motivational and material concerns of OVC, they can function effectively within their school environment and be better equipped to complete their education (Mbatha, 2014). When addressing the educational needs of OVC, it is important to ensure that physical needs are addressed, support is provided with regard to schoolwork, and to alleviate the financial stress of caregivers with regard to receiving education (Mutenheri, 2014). Assisting both orphans and caregivers with regard to material needs plays a crucial role in allowing OVC to complete their education. The sustainability of interventions is impeded when there are insufficient resources available to address the material needs of OVC and when the interventions only focus on their material needs (Mutenheri, 2014), thereby limiting the opportunities for orphans to function optimally both within and outside of schools (Mutenheri, 2014).

### Psychoeducational interventions

Psychoeducational interventions were the third most frequently implemented intervention and were described in six of the 17 articles analysed. Psychoeducational interventions described in the literature were restricted in terms of their variety and how these interventions were implemented. Psychological disorders, abuse and neglect, and HIV/AIDS were some of the challenges identified that OVC face in South Africa (Allman et al., 2022). As a result, it is essential to provide OVC and caregivers with the necessary education about these challenges in order to improve their psychological and physical well-being (Pillay, 2018). Most psychoeducational interventions in this category included elements pertaining to the education of HIV/AIDS and related aspects. Therefore, to avoid repetition, only aspects unique to the interventions will be discussed.

#### Geographical spread of interventions

Psychoeducational interventions were provided to OVC in Gauteng, the Eastern Cape, Kwazulu-Natal, and Mpumalanga. This reach indicates that psychoeducational interventions were implemented among 61.8% of South African orphans (Hall, 2019). Considering the background from which OVC originated, in addition to the numerous psychological disorders and sexual diseases that OVC are at risk of, this raises concerns with regard to the psychoeducational support provided to OVC with regard to these matters. Furthermore, some interventions were limited in that they only provided support to certain types of OVC, and service delivery was restricted to specific areas within the provinces. As a result, it is possible that the percentage of OVC that received psychoeducational support might have been even smaller.

#### Types of psychoeducational interventions provided

Psychoeducational support was provided to OVC in the forms of: (a) focusing on prosocial behaviours, HIV/AIDS education, and safe sex practices, addressing behavioural problems, and interpersonal relationships, improving their quality of life (Sitienei & Pillay, 2019), (b) life skills training to OVC on HIV, its risks, prevention thereof, and substance abuse education (Visser et al., 2015), (c) informational support on HIV/AIDS, safe sex practices, and other health-related concerns of OVC (Kwatubana & Ebrahim, 2020), (d) peer-based educational support with regards to understanding concepts of death and dying (Thupayagale-Tshweneagae & Mokomane, 2014), (e) addressing HIV risk factors, alcohol and substance abuse, crime, sexual violence, and condom use (Thurman et al., 2016a, 2016b), and (f) skill training and education with regards to HIV/AIDS by means of support groups (Marais et al., 2013).

Despite the variety of psychoeducational interventions provided to OVC, there are numerous challenges and limitations that impede their effectiveness.

#### Challenges and limitations of interventions

The provision of life skills to OVC is essential because these skills allow them to gain knowledge, attitudes, and skills that can enable them to behave in a healthy manner (Nyathi, 2022). As orphans are highly susceptible to behavioural problems, poor interpersonal relationships, have low levels of social support, and are more likely to contract HIV/AIDS, the provision of education and skills pertaining to prosocial behaviours, HIV/AIDS education, and safe sex practices, addressing behavioural problems, and interpersonal relationships are essential (Breckenridge et al., 2019; Sitienei & Pillay, 2019). However, the efficacy of these interventions is subject to the social and environmental factors wherein OVC function (Ghirwa, 2014). When taking into consideration that these factors often do not allow for OVC to feel supported, supplementary support should be provided to OVC when receiving life skills training and education (Ghirwa, 2014).

Preventing substance abuse is crucial, especially given the lack of financial means for rehabilitation services and the numerous physical and mental health issues associated with substance use (Meghdadpour, Curtis, Pettifor, & MacPhail, 2012). Unfortunately, psychoeducational strategies for addressing substance abuse not as effective as other therapeutic treatment methods for those who are already addicted (López, Orchowski, Reddy, Nargiso, & Johnson, 2021). As a result, OVC who are already addicted to a substance may not benefit from these types of interventions.

It is important to emphasise the importance of allowing OVC to share their health concerns as OVC generally have a variety of health concerns (Kwatubana & Ebrahim, 2020). Another concern to address is the concepts of death and dying among orphans, and it is important to ensure that they have an accurate understanding of these concepts (Thupayagale-Tshweneagae & Mokomane, 2014). To do so, OVC need to develop constructive coping mechanisms and thereby share their experiences and express their needs (Thupayagale-Tshweneagae & Mokomane, 2014). These forms of interventions are crucial as OVC experience severe emotional distress after the death of their parent/s, and typically results in negative coping mechanisms (Ntuli et al., 2020). However, given the fact that majority of OVC are not situated in supportive environments wherein their expressed feelings and concerns will be heard and addressed (Ntuli et al., 2020), this may limit the effectiveness of interventions teaching orphans these set of skills.

### Physical Resource Interventions

Analysis revealed that psychoeducational interventions were the fourth most frequently implemented intervention and were used in five of the 17 articles analysed. According to STASSA (2020), 51% of South African children are monetarily poor; that is, their monthly consumption is below the lower-bound poverty line of R647 per person per month. As a result, OVC and caregivers struggle to obtain the physical materials required for survival (Ntuli et al., 2020). Therefore, having interventions in place to address the physical needs of OVC and their caregivers is crucial to improving their quality of life.

#### Geographical spread of interventions

Physical resource interventions were provided to OVC in Gauteng, the Free State, Western Cape, Kwazulu-Natal, Eastern Cape, and Mpumalanga. This means that 86.6% of OVC in South Africa received physical resource support. However, the issue pertaining to service delivery coverage and the type of OVC that received services remains. As a result, this could mean that the actual percentage of OVC who received physical resource support was less than mentioned. This indicates that there were still OVC who were not receiving physical resource support in South Africa.

#### Types of physical resource interventions provided

Physical resource support was provided in the following ways: (a) providing gardening support to OVC caregivers (Eale, 2018), (b) providing OVC with safe parks to play in and alternative care environments (Marais et al., 2013; Mutenheri, 2014; Visser et al., 2015), and (c) providing HIV testing materials (Thurman et al., 2016a, 2016b). However, despite the variety of physical resource interventions provided to OVC, there are numerous challenges and limitations that impede their effectiveness.

#### Challenges and limitations of interventions

Malnutrition is prevalent among OVC and caregivers in South Africa, necessitating nutritional support (Eldah, Mary, Selina, & Cecilia, 2022). None of the interventions provided nutritional education, and most were not sustainable due to resource limitations. Because OVC typically experience physical and sexual abuse, it is important to implement interventions that provide OVC with a safe place to play or alternative care environments, such as children's homes, temporary shelters, and daycare facilities (Visser et al., 2015). HIV/AIDS is also a prevalent occurrence among OVC. To address this challenge, physical resource interventions that focus on providing HIV testing material are crucial if we want to improve HIV testing uptake and treatment within resource-constrained areas (Thurman et al., 2016a, 2016b).

### Service interventions

Analysis revealed that service interventions were the fourth most frequently implemented intervention and were described in five of the 17 articles analysed. OVC and family members or caregivers typically do not have access to the resources required to improve their quality of life (Mutiso & Mutie, 2018). As a result, OVC and their caregivers rely on external sources of support and services; however, OVC and caregivers typically do not have access to these services or receive poor provision of these services (Mutiso & Mutie, 2018). Therefore, it is important that interventions are in place to give OVC and caregivers access to these services.

#### Geographical spread of interventions

The literature study showed that such service interventions were provided to OVC in Kwazulu-Natal, Eastern Cape, Free State, Mpumalanga, and Gauteng. This means that 77.7% of OVC in South Africa would have access to service interventions (Hall, 2019). Complicating this percentage is the fact that service delivery from some of these interventions was only conducted in certain areas of the provinces, and some interventions were only provided to certain types of OVC. Therefore, the actual number of OVC who received service interventions may have been lower.

#### Types of service interventions provided

Service interventions were provided in the following ways: (a) providing referrals for OVC and caregivers to governmental and community services such as education, health care, social services, birth registration, child grants, and foster care (Visser et al., 2015), (b) providing referrals to similar services mentioned before in addition to obtaining access to food parcels and nutritional supplements (Marais et al., 2013; Mutenheri, 2014), and (c) making referrals to health and social services to promote HIV testing endeavours among OVC (Eale, 2018; Thurman et al., 2016a, 2016b).

Despite the numerous service interventions provided to OVC, there are numerous challenges and limitations that impede their effectiveness.

#### Challenges and limitations of interventions

OVC and caregivers typically require referrals to more than one service from numerous sectors due to the needs they present with (Stover et al., 2007). Therefore, intersectoral collaboration is required for these referrals to be successful in aiding OVC. The challenge is that there is a lack of intersectoral collaboration within South Africa because of a lack of support between sectors, funding constraints, and problems with role delineation (Brooke-Sumner, Lund, & Petersen, 2016). This typically results in service provision being delayed or not received at all and limits the amount of aid that can be provided by service referral efforts from organisations (Brooke-Sumner et al., 2016).

South Africa's health service sector has received a great deal of scrutiny due to resource scarcity and poor management (Maphumulo & Bhengu, 2019). Despite providing free healthcare, these institutions are difficult to reach and often lack necessary medicine, leading to OVC and caregivers being referred to other institutions and discouraged from visiting hospitals (Mutiso & Mutie, 2018). South Africa's social grant services have also received scrutiny, with caregivers often not receiving adequate support for their needs and the needs of OVC. Challenges like obtaining birth certificates and identity documents further hinder their access to eligible social grants (Nzuza, 2020).

### Emotional interventions

Analysis revealed that emotional interventions were the fourth most frequently implemented intervention and were described in five of the 17 articles analysed. According to Mutiso and Mutie (2018), OVC are especially prone to experiencing emotional problems because of being deprived of the love and care typically received from family members. This results in OVC having low life satisfaction and affects their psychological well-being in addition to their general quality of life (Raats, Adams, Savahl, Isaacs, & Tiliouine, 2018). Therefore, it is important that mechanisms be set in place to allow orphans to receive emotional support to improve their satisfaction with life and psychological well-being.

#### Geographical spread of interventions

Emotional interventions were provided to OVC in Gauteng, Eastern Cape, and KwaZulu-Natal. This indicates that 48% of OVC would have received emotional interventions in South Africa (Hall, 2019). This is concerning as it also means that more than half of South African OVC did not receive emotional support. Furthermore, given the fact that service delivery distribution varied within each province and that only certain types of OVC received emotional support, this percentage of OVC reached could have been even less. When taking into consideration the fact that OVC are susceptible to a multitude of emotional disturbances because of their background, death of caregiver/s, and being exposed to institutional care and experiences associated with being an orphan, this is an issue that needs to be addressed through more widespread emotional intervention implementation.

#### Types of emotional interventions provided

OVC received emotional interventions in the forms of: (a) social workers and pastors addressing the emotional needs of OVC because of the death of their parent/s (Kwatubana & Ebrahim, 2020), (b) Interpersonal psychotherapy groups (IPTG) to promote OVC involvement in each other’s therapeutic process (Thurman et al., 2016a), (c) cognitive behavioural therapy (CBT), psychoeducational strategies, and quality caregiver interactions (Breckenridge et al., 2019), and (d), quality caregiver interactions alone (Balie & Sayed, 2020; Mbatha, 2014).

Despite the variety of emotional interventions provided to OVC, there are numerous challenges and limitations that impede their effectiveness.

#### Challenges and limitations of interventions

Connolly et al. (2021) suggested that social workers and pastors functioning as lay counsellors can be an effective means of providing emotional support through enhancing coping abilities and thereby improving their ability to recover from the loss of their parents. However, this is only true when individuals receive sufficient training and support as lay counsellors and when they involve further support mechanisms, namely registered professionals when a situation calls for it. IPTG can be an effective means of reducing anxiety and depression symptoms, but only if cases are not severe (Thurman et al., 2016a). In severe cases, pharmacological interventions may be required, but these pose a challenge with regard to obtaining access to medication. Therefore, referral and support mechanisms should be in place to provide support to OVC who require such interventions.

CBT can help reduce symptoms of depression, anxiety, anger, trauma, and low self-esteem (Isnaeni et al., 2021). Where psychoeducational strategies improve self-awareness and emotional development, it is quality caregiver interaction that is crucial for a child's overall growth, ensuring optimal development (Lovino, Caemmerer, & Chafouleas, 2021). The proper caregiver interaction can allow OVC to develop self-awareness of their emotional state and the factors that influence it, thereby ensuring optimal emotional development (Cejalvo, Martí-Vilar, Merino-Soto, & Aguirre-Morales, 2021). Allowing teachers to function in a capacity in which they can address the emotional needs of OVC can be very advantageous as it allows for quality caregiver interactions to occur, provided that the teachers receive sufficient training in identifying and addressing the emotional needs of OVC (Balie & Sayed, 2020; Mbatha, 2014). However, teachers should also have the required resources available to them if referrals are required in severe cases. Therefore, an appropriate referral structure should be in place containing the details of individuals in the community who can provide those services to OVC.

### Behavioural interventions

Analysis revealed that behavioural interventions were the fifth most frequently implemented intervention and were described in three of the 17 articles analysed. Ntuli et al. (2020) found that South African children in orphanages displayed deficits in their behavioural regulation abilities.

Where Ntuli et al. (2020) stated that South African orphans face numerous behavioural challenges these related to: (a) managing relationships with authority figures, (b) completion of academic tasks, (c) poor academic performance, and (d) adhering to a code of conduct (sets of rules within orphanages or at school). Therefore, the behavioural needs of orphans need to be attended to as they affect numerous areas of functioning (Ntuli et al., 2020).

#### Geographical spread of interventions

Behavioural interventions were provided to OVC in the Free State, Eastern Cape, and Western Cape. This indicates that 44.6% of South African orphans could have received behavioural support (Hall, 2019). However, given the fact that service provision was restricted to certain areas in these provinces and that certain interventions focussed on specific types of OVC, this percentage may have been lower. Given the prevalence of behavioural disturbances among OVC and the far-reaching implications these have on their academic and interpersonal functioning, lack of access poses a concern that needs to be addressed by providing more widespread behavioural support.

#### Types of behavioural interventions provided

Behavioural interventions were provided in the forms of: (a) preventative and modification programmes (Marais et al., 2013), (b) psychoeducational and cognitive behavioural strategies (Breckenridge et al., 2019), and (c) lay counselling techniques to improve relationships between teachers and OVC (Balie & Sayed, 2020). Despite the variety of behavioural interventions provided to OVC, there are numerous challenges and limitations which impede their effectiveness.

#### Challenges and limitations of interventions

The prevention and or modification of behavioural disturbances among OVC are crucial in that the majority of OVC originate from a background wherein behavioural disturbances are already present or occur because of exposure to the experience of becoming an orphan (Doku et al., 2019). As a result, both the prevention and modification of behavioural disturbances is crucial in the process of enabling OVC to develop optimally (Cluver et al., 2012; Doku et al., 2019). However, for these efforts to be effective, Zhang et al. (2022) recommended that the material needs of OVC need to be addressed in addition to providing counselling and motivational support. As a result, cognitive behavioural and psychoeducational strategies can be effective in producing behavioural improvements and could result in improved prosocial behaviours (Breckenridge et al., 2019). Another important aspect to consider is the quality of caregiver interactions that OVC receive (Warner et al., 2017). Through implementing lay counselling techniques, the behavioural challenges of OVC can be mitigated because of the positive relationship between OVC and caregivers if adequate training is provided in lay counselling techniques (Balie & Sayed, 2020; Warner et al., 2017).

### Family interventions

Family interventions were the fifth most frequently implemented intervention and were described in three of the 17 articles analysed. Organisations play a crucial role in providing support to families of OVC, as they often face emotional distress, limited social support, and frequent family disputes (Pillay, 2018). Providing support to family members of OVC enhances their ability to provide quality caregiver interactions, thereby enhancing the psychological and physical development of the OVC and general quality of life (Pillay, 2018).

#### Geographical spread of interventions

Family interventions were provided to OVC in Gauteng, Kwazulu-Natal, Eastern Cape, and Mpumalanga. This indicates that 61.3% of South African OVC could have received family support (Hall, 2019). However, this percentage may have been lower because service delivery was only conducted in certain areas of these provinces, and some interventions were only provided to certain types of OVC. Given the difficulties caregivers of OVC typically experience because of poverty or lack of access to resources, in addition to the effects this has on OVC, the lack of interventions raises a concern that needs to be addressed by providing more widespread family support that comprehensively identifies and addresses the needs of the caregivers of OVC.

#### Types of family interventions provided

OVC received family support in the following ways: (a) aiding in the payment of rent and providing monthly food supplies (Sitienei & Pillay, 2019), and (b) facilitating open communication between OVC and caregivers (Eale, 2018; Visser et al., 2015). From the literature, it can be observed that only a limited amount of family support is provided to OVC and caregivers. As a result, there are numerous challenges and limitations which impede the effectiveness of these interventions especially when considering the vast number of needs that both OVC and caregivers present with.

#### Challenges and limitations of interventions

Caregivers of OVC are typically not financially capable of caring for OVC and face numerous challenges with regard to housing, food, education, and health care (Mwinzi, Kathuri, & Kinzi, 2020). This results in caregivers not being able to provide the required resources to OVC to ensure that they are multidimensionally cared for (Mwinzi et al., 2020). Although governmental and non-governmental organisations address the needs of OVC, caregivers still require support as they are unable to address their own needs (Ojo & Olayinka, 2019). As a result, intervention efforts that provide aid to both OVC and caregivers are essential to allow for the optimal and sustainable development of OVC (Ojo & Olayinka, 2019).

Improving open communication between OVC and caregivers is essential for the development of strong and positive relationships (Procentese, Gatti, & Di Napoli, 2019). Open communication can improve family functioning and lead to an increase in family support and improvement in the well-being of all involved (Procentese et al., 2019; Visser et al., 2015). However, it is important that service providers facilitate open communication regularly and not only in times when the family dynamics are in dire need of intervention (Eale, 2018; Visser et al., 2015).

### Developmental interventions

Developmental interventions were the sixth most frequently implemented intervention, as described in one of the 17 articles analysed. OVC often experience significant developmental delays with regard to physical growth, hormonal development, cognitive development, and emotional development, and these delays are often the result of insufficient physical resources or caregiver interactions (van IJzendoorn et al., 2011). As a result, it is especially important for institutions to pay attention to the developmental delays presented by OVC to specifically address them, thereby promoting optimal development (van IJzendoorn et al., 2011).

#### Geographical spread of interventions

Developmental interventions were only provided to OVC in the Free State, Eastern Cape, Kwazulu-Natal, and Limpopo. This indicates that 62.3% of South African OVC could have received developmental support (Hall, 2019). However, this percentage could be far less given the fact that this type of intervention was only present within one study and was only provided within certain service delivery sites in the provinces. Furthermore, this study only focussed on street children, which could further reduce this percentage.

#### Types of developmental interventions provided

Developmental interventions were provided by ensuring that children were placed in safe and functioning homes, focusing on positive relationships between OVC, caregivers, and shelter workers, and educating OVC on aspects of basic hygiene and nutrition (Mokomane & Makoae, 2015). Despite the variety of means through which developmental interventions were provided OVC, there are challenges and limitations that impedes their effectiveness.

#### Challenges and limitations of interventions

When taking into consideration the fact that OVC are more likely to experience developmental delays when compared to non-orphaned children, the need for more wide-spread service delivery is required. Additionally, it is important that the exact developmental needs of the OVC be taken into consideration when attempting to address these needs (Housman, 2017). Family relationships, good hygiene practices, and a positive educational environment significantly impact a child's development. Furthermore, positive family relationships facilitate optimal growth, while good hygiene practices aid in physical development and cognitive, social, and emotional growth (Thomas et al., 2020; van IJzendoorn et al., 2011). Although the shelters try to provide OVC with the needed exposure, knowledge, and environment for optimal development, the efficacy remains uncertain (Mokomane & Makoae, 2015). Shelters ought to network with implicated role players to ensure that age-appropriate care can be provided (Mokomane & Makoae, 2015).

### Recreational interventions

Recreational interventions were the sixth most frequently implemented intervention, as described in one of the 17 articles analysed. Recreational activities enhance the social well-being of children by teaching them valuable skills required to form lasting bonds and fostering reciprocal relationships, making them more adept at making and maintaining friends (Hoffer, n.d.)

#### Geographical spread of interventions

Recreational interventions were provided to OVC in the Free State, Eastern Cape, Kwazulu-Natal, and Limpopo. This indicates that 62.3% of South African OVC could have received recreational support (Hall, 2019). However, this percentage could have been far less given that this type of intervention was only present in one study and was only provided in certain service delivery sites in the provinces mentioned. Furthermore, this study only focussed on street children, which could further reduce this percentage.

#### Types of recreational interventions provided

The recreational intervention provided included providing OVC with board games, allowing OVC to watch television, and educating OVC with regard to arts and crafts skills (Mokomane & Makoae, 2015).

#### Challenges and limitations of interventions

Recreational facilities were in sub-standard condition and were exclusive to street children (Mokomane & Makoae, 2015). This resulted in little to no socialisation with other children in the community and further placed scrutiny on the efficacy of this intervention in improving the social well-being of OVC (Mokomane & Makoae, 2015). Furthermore, an extremely limited number of recreational options were provided to OVC.

### Resilience interventions

Resilience interventions were the sixth most frequently implemented intervention in the 17 articles. OVC face challenges in developing resilience due to limited resources because of economic, psychological, and social barriers (Makhonza, 2018). These barriers, especially those originating from a traumatic background, hinder their ability to harness positive coping mechanisms and maintain psychological equilibrium (Makhonza, 2018).

#### Geographical spread of interventions

Resilience interventions were provided to OVC at two service delivery sites in South Africa. The study did not indicate which province the support was provided to. However, given the fact that this support was only provided at two service delivery sites and the traumatic background from which OVC originated, resilience is crucial for OVC, and more widespread service delivery is required (Mutiso & Mutie, 2018).

#### Types of resilience interventions provided

Resilience support was provided to OVC through the Memory Book intervention, which aimed to improve the resilience of OVC by facilitating the process of overcoming the traumatic loss of caregivers (Braband et al., 2018). From this it can be concluded that very limited resilience interventions are provided to OVC, which poses its own set of challenges. Furthermore, the intervention implemented presented with its own set of challenges and limitations.

#### Challenges and limitations of interventions

By focusing on personal resources, the intervention facilitated the development of positive coping mechanisms, increased hope, and positive emotions (Braband et al., 2018). However, the intervention only focused on personal resources and not the physical resources, which could have contributed to the OVC becoming more resilient by providing physical buffers (Braband et al., 2018). Age and gender are also important variables with regard to resilience and should be taken into consideration when implementing such interventions (Priyadarshini & Rathnasabapathy, 2021).

### Language literacy interventions

Language literacy interventions were the sixth most frequently implemented intervention in the 17 articles. Children's reading and writing skills are crucial for academic success, especially in impoverished rural areas (Stockard, 2011). Ensuring age-appropriate reading and writing skills is crucial for orphans from impoverished backgrounds (Stockard, 2011).

#### Geographical spread of interventions

Language literacy interventions were only provided to OVC in Kwazulu-Natal, which indicates that only 15.6% of OVC in South Africa could have received language literacy support. However, given the fact that this intervention was only provided to OVC from one site in Kwazulu-Natal, this percentage could be lowered further. The provision of language literacy support is crucial in ensuring that OVC have basic reading and writing skills, considering that OVC are more likely not to obtain or complete basic education (Bachore, 2022). When taking the aforementioned and the fact that this intervention type was only provided at one intervention site, numerous challenges and limitations are present which hinders the effectiveness of the intervention.

#### Types of language and literacy interventions provided

OVC received language literacy support through volunteers and therapists who provided language and literacy support to OVC (Zoetmulder, 2019). Volunteers were essential as language and speech therapists are limited in South Africa. Volunteers were able to improve the reading and writing skills of OVC through training and collaboration with therapists (Zoetmulder, 2019).

#### Challenges and limitations of interventions

With the vast number of OVC within South Africa who present with deficits in their language and literacy skills and the limited number of trained professionals to address this, further investment from governmental and non-governmental organisations are required to ensure that enough individuals are appropriately trained. This is to ensure that OVC receive the support required while providing long-term support as intervention efforts of this nature requires long-term involvement for them to be successful (Zoetmulder, 2019).

## Limitations of the study

The study only included literature obtained from three chosen databases. Furthermore, review studies and books or chapters of books were excluded. Only academic literature was collected for this study, therefore excluding the possibility of interventions that were implemented in a situation where no research studies were conducted. Only 17 articles were included for analysis. Considering these limitations, caution should be applied when concluding the results of the study. Additionally, due to the limited number of articles included, the results of the study should not be considered representative of all interventions currently implemented among South African orphans.

## Conclusion

The studies included in this scoping review indicated that there were numerous types of interventions implemented among orphans within South Africa. They were provided by a variety of organisations at the time of the study. It was clear that variations were present with regard to how the different types of support were provided to orphans. The majority of the studies indicated that these interventions and associated methods were successful in improving OVC well-being. However, the majority of the studies also indicated that there were numerous barriers present that inhibited the interventions’ ability to fully improve the well-being of OVC. Sustainability and longitudinal impact of interventions were frequent concerns, in addition to intervention efforts that only addressed a specific aspect of the needs presented by OVC. Several recommendations have been made to help implement a balanced approach wherein the basic physical needs of OVC can be addressed first, followed by their psychological needs. It is recommended that intervention efforts should first identify the specific needs of OVC and address these needs in a balanced approach rather than providing support to only a specific aspect of needs presented by OVC.

## Supplementary Material

Appendices.docx

## Data Availability

All studies used within this scoping review are available in open access in the databases provided in this study.

## Appendix

**Table A1.** Data extraction table of eligible studies.

No.	Title of article	Author(s) and date of publication	Methodology	Participants	Findings or Results	Conclusion
1.	Psychosocial Support for Orphaned and Vulnerable Children with HIV/AIDS in Eastern Cape, South Africa.	Mufalali, R. M., Makua, M. G. & Matlhaba, K. L. (2022).	A descriptive qualitative design of appreciative enquiry.	30 OVC aged between 13 and 17 located in the Maluti sub-village.	Thanks to the South African Red Cross Society (SARC) Maluti project, the OVC were able to develop positive coping mechanisms due to the provision of incentives, education, indoor and outdoor activities, and gardening skills. These also allowed the children to develop close bonds with fellow peers and caretakers. Children also felt that the intervention addressed their developmental, social, and mental needs. The children also appreciated the small group or individual treatment that was given to them.	The SARC Maluti project has been successful in supporting OVC with regard to their psychosocial needs. Furthermore, OVC can thrive when their basic psychosocial needs are met, which highlights the importance of ensuring that these needs are met.
2.	Psycho-educational and social interventions provided for orphans and vulnerable children at a community-based organisation in Soweto, South Africa.	Sitienei, E. C. & Pillay, J. (2019).	Qualitative: a phenomenological approach.	12 OVC in Soweto were selected by a community-based organisation focussing on OVC exposed and affected by AIDS. Where five were males and seven were females.	The study identified psychological, educational, and social interventions to be present. Findings suggest that OVC gain significantly from mentorships and peer-group support regarding psychological support. However, it was also found that some participants (*n* *=* *2*) found it difficult to trust mentors or peers. Educational interventions entailed the provision of educational material, food hampers, payment of tuition fees, and payment of excursions and sports activities. Social interventions entailed the OVC learning life skills and receiving financial support for their families. However, the sustainability of the financial support provided is uncertain.	The study concluded that, although the intervention efforts are successful, there is a need for comprehensive support networks to address psychological and social issues. Additionally, counselling services and income-generating projects ought to be a focus within OVC communities affected by HIV/AIDS.
3.	ISIBINDI, creating circles of care for orphans and vulnerable children in South Africa: post-programme outcomes.	Visser, M., Zungu, N. & Ndala-Magoro, N. (2015).	Mixed methods: quasi-experimental design with focus group discussions (FGD).	A total of 604 OVC were chosen as participants and were identified from all twelve sites of the ISIBINDI community-based intervention. These sites are situated in Kwazulu-Natal, Eastern Cape, Mpumalanga, and Gauteng. Participants included 427 ex-participants of the intervention and 177 control group members. All participants were 18–25 years old.	The ISIBINDI project provided support in the following ways: (1) home visits and family support, (2) personal guidance and counselling, (3) access to a safe park, (4) provision of aid with regards to education and career guidance, (5) life skill training, and (6) access to health care and treatment. Quantitative analysis indicated that ex-participants of the intervention had higher mean self-esteem scores when compared to the control group (M = 5.01 and 3.82, respectively; p-value <.01). Ex-participants also had higher mean problem-solving skills when compared to the control group (M = 3.97 and 2.26 respectively; p-value <.05). Family support was also higher in ex-participants when compared to control groups (M = 4.62 and 3.31 respectively, p-value <.05). Ex-participants also had lower HIV-risk behaviours when compared to the control group (M = 12.9 and 19.7 respectively, p-value <.05). Qualitative results indicated overall improvement in psychological well-being; especially pertaining to self-esteem, problem-solving skills, and family communication and relationships.	The evaluation of the ISIBINDI programme found that this intervention, on a multi-faceted level, provided positive outcomes that allowed for protective barriers to OVC.
4.	Psychosocial Support Provision for Learners from Child-Headed Households in Five Public Schools in South Africa.	Kwatubana, S. & Ebrahim, M. (2020).	Qualitative: a phenomenological approach.	Participants consisted of principles (*n* *=* *5*), teachers (*n* *=* *9*), and health programme coordinators (*n* *=* *5*) from five schools in the Sedibeng East district. Nineteen participants were selected based on who attended to the most Child-Headed Household (CHH) students.	Results of the study indicated that CHH students received material, emotional, and spiritual support from various sources. With regards to physical and material needs, the National School Nutrition Programme, uniform programme, Sanitary Pads Project, Food Garden Project, Nature’s Choice, and school programmes involved with the provision of adult supervision were present. These programmes and projects aided CHH individuals with food, school uniforms, and adult supervision in various ways. With regards to emotional and psychological support, all schools had social workers who frequently made visits to the school for counselling sessions, in addition to one school that had counsellors and nurses from Lifeline educate students on HIV/AIDS and teenage pregnancy. Furthermore, two of the schools arranged for pastors to visit their schools and provide guidance to students. All programmes, projects, and interventions originated from either governmental or non-governmental organisations.	The study found that although a variety of interventions were present, their efficacy remained uncertain. Furthermore, there is a need for schools to change their health policies and school-wide practices to be more accommodating of interventions that can target CHH students specifically.Teachers also require further training on trauma-sensitive practices as well as stress, burnout, and secondary trauma when working with children from CHHs. Additionally, more collaboration between teachers and social workers is required to ensure that a multipronged approach can be feasible.
5.	Psychological and behavioral interventions to reduce HIV risk: evidence from a randomized control trial among orphaned and vulnerable adolescents in South Africa.	Thurman, T. R., Kidman, R., Carton, T. W. & Chiroro, P. (2016a).	Quantitative: randomised control trail.	Students aged 14 –17 years old enrolled in the World Vision South Africa community-based Networks of Hope OVC programme. The Networks of Hope programme offers two interventions: (1) interpersonal psychotherapy for groups and (2) a curriculum-based behavioural intervention. Students were in 84 villages in two districts in the Eastern Cape. This equated to 1,016 participants.	Interpersonal psychotherapy for groups focused on improving interpersonal skills and providing emotional support. Whereas the Vhutshilo intervention focused on behavioural changes by addressing topics such as alcohol and substance abuse, crime and sexual violence, HIV/AIDS, healthy sexual relationships, transactional sex, and condom use. The review found that only significant intervention effects were present when individuals participated in both interventions. Furthermore, effects also varied by gender. For girls, condom use increased by approximately 32% when compared to baseline survey results. Whereas in the control group, only a 7% increase in condom use occurred. With regards to boys, the prevalence of risky sexual behaviour was significantly lower when compared to the control group. Furthermore, the predicted probability of engaging in risky sexual behaviour increased by 9% in boys from the control group. In comparison, the predicted probability of engaging in risky sexual behaviour stayed the same for boys who participated in both interventions.	The study concluded that, although the interventions were effective, they functioned in conjunction with existing interventions that provided aid for economic and educational needs. The study also concluded that community-based interventions that rely on local community members and deliver services directly to OVC and their households are effective and provide a promising model for other interventions. Additionally, the combination of theory-driven psychological and behavioural intervention packages is promising and should be promoted amongst OVC.
6.	Community-based mental health support for orphans and vulnerable children in South Africa: a triangulation study.	Marais, L., Sharp, C., Pappin, M., Rani, K., Skinner, D., Lenka, M., Cloete, J. & Serekoane, J. (2013).	Mixed methods.	The study consisted of two sets of participants. One set was management and staff members of community-based organisations that serve OVC within the Mangaung Municipality, Free State. The second set consisted of 607 OVC from the same area.	Findings showed that the community-based organisations had eight aims aligned with those the Free State Provincial Government laid out. These aims: (1) the provision of care services, (2) the provision of support services, (3) attending to psychological needs, (4) behavioural prevention and modification programmes, (5) the provision of training programmes, (6) provision of social assistance, (7) community mobilisation, and (8) provision of care facilities. Qualitative results indicated that all the CBOs in the area had different conceptualisations of their purposes. However, all the CBOs agreed that alleviating poverty was their most important aspect. Quantitative analysis indicated that only three of the aims had a positive statistically significant relationship with the scores obtained on the Strengths and Difficulties Questionnaire (SDQ). Access to medical services, food expenditure and total expenditure were related to positive mental health outcomes.	The study produced four conclusions. First, funding guidelines provided by the government influence how CBOs position themselves regarding their purpose. Second, CBOs are mainly there to help the government carry out what the government considers to be its own work. Third, guidelines make little mention directly of the mental health of OVC and that HIV/AIDS is more a socio-economic problem. Fourth, cash transfers were not an effective means of providing intervention.
7.	Educational support for orphans and vulnerable children in primary schools: Challenges and interventions	Mwoma, T. & Pillay, J. (2015).	A mixed methods approach involving descriptive and qualitative designs.	Participants were teachers and Grade 7 students from public primary schools in Soweto. A total of 107 individuals participated in the study, with 42 being teachers and 65 being students (43 boys and 22 girls).	The government provided Interventions to pay OVC’s school fees in addition to providing their books and stationery. Furthermore, governmental soup kitchens provided OVC with meals. External organisations were involved in providing students with school uniforms, while teachers played an active role in motivating and aiding OVC students who struggled academically. Although most of the OVC students corroborated the above-mentioned provisions, there is a small percentage of OVC students not receiving these services. Teaches reported that they do not have enough time to attend to the needs of OVC students, with the time pressure to finish curriculum work with students in addition to working long hours. OVC students displayed many challenges. They were marginalised by peers and other teachers, had reading, and writing difficulties, had low self-esteem, often displayed mood disorders and had difficulties concentrating in class and on schoolwork. Furthermore, OVC students also had greater rates of absenteeism or lateness in school attendance. With regard to meal provision, both students and teachers felt that the needs of the OVC far exceed the available resources. OVC students would often come to school hungry as there is no food at home, which poses another problem in that meal provisions can only be done for students and not their family members.	Although the government has put in place numerous means by which OVC can be aided, there are still OVC needs that are unmet. Teachers require further training with regard to engaging with so many OVC. Food programmes ought to be implemented at the homes of the OVC, and education is needed among parents and guardians pertaining to the importance of school attendance and homework completion by the OVC.
8.	Evaluation of a peer-based mental health support program for adolescents orphaned by AIDS in South Africa.	Thupayagale-Tshweneagae, G. & Mokomane, Z. (2014).	Qualitative: phenomenological approach with focus group discussions.	Participants were 15 adolescents aged 14–18 who were orphaned as a result of AIDS and who participated in the Better Accept Reality (BAR) programme.	During the evaluation process, it was noted that most of the participants became more conversive with regard to discussing death and dying and coming to terms with accepting the death of their parent or parents. Listening skills and expression of thoughts also improved among most participants. Furthermore, most of the participants also become more assertive about their needs, thoughts, and feelings. Additionally, problem-solving skills also improved for most of the participants. Participants also found that their relationship with their caregivers had improved, although some reported that it was too early for them to respect their caregivers after being treated so poorly by them. The final observation was that some participants improved their school grades; however, this was a small number of participants (*n* *=* *4*).	The study findings indicated that the BAR programme effectively improves participants’ psychological well-being. Furthermore, the reorientation of peer-based mental health programmes for adolescents orphaned as a result of AIDS ought to be considered because psycho-educational components can help improve the mental health of these individuals. Furthermore, these programmes should be implemented at schools. Aspects such as family support and caregiver commitment ought to be taken into account when orphan placement is considered.
9.	HIV/AIDS orphans in South Africa: NGO interventions supporting transitions to alternative care.	Breckenridge, T. A., Black-Hughes, C., Rautenbach, J., & McKinley, M. (2019).	Qualitative: phenomenological approach.	Forty-nine orphans in a small rural village in the Eastern Cape.	The NGO interventions employed psychoeducational and cognitive behavioural strategies. The study found themes pertaining to the bereavement displayed by children towards their parent/s death or absence, a need for physical or tactile attention, negative behaviours at the beginning of the intervention and an overall improvement at the end of the intervention. Furthermore, differences were found in children who were orphaned due to different causes (due to HIV/AIDS, unknown or abandonment). With regards to bereavement, most of the children displayed sadness and crying; some were not aware of their parents’ death, some experienced guilt, some refused to talk about their parents’ death, denial, anger, worry, and were haunted by the death of their parent/s, scared, some ran away from their caregivers, some blamed others, shyness and isolation. With regards to touch and affection, six required physical and tactile attention. Of these six children, three required affirmation that they are loved. Furthermore, only eight of the OVC displayed happiness and satisfaction with their caregivers. Psychoeducational support provided to the children, community and caregivers allowed for improvement pertaining to mood and interpersonal relationships and interactions within 35 of the OVC. Furthermore, the children found it easier to play with others, express themselves and showed improvement in behaviours.	The NGO focused on developing healthy lifestyles, providing leadership skills and daily living skills, providing education, and enhancing self-esteem. Destructive and low self-esteem behaviours declined over six months. Additionally, the study showed that OVC requires interventions that tap into their social, educational, and psychological needs.
10.	An overview of programmes offered by shelters for street children in South Africa	Mokomane, Z. & Makoae, M. (2015).	Qualitative approach with semi-structured interviews.	Social workers, shelter managers, and the provincial and district authorities are responsible for the implementation of the Children’s Act and Norms and Standards. The individuals were from two districts situated in four provinces in South Africa (two per district, per province).	The study found that all shelters in all the study districts adhered to the Children’s Act and Norms and Standards. Furthermore, all the shelters provided interventions in the form of developmental, recreational and therapeutic programmes.	The study concluded that current national legislative and policy frameworks in South Africa align with the rights and protection afforded to street children internationally. Furthermore, the programme focuses on early intervention and can be deemed an appropriate means of providing aid to children. A point of contrition, however, is that all shelters predominantly focus on psychosocial support and life skill training as a means of altering children’s behaviours. There is also a widespread emphasis on enrolling street children into local mainstream schools that cannot attend to their needs pertaining to developmental delays. Programmes offered by shelters are also centred around children and exclude other key stakeholders. As a final point, despite the comprehensive legislative and policy frameworks, the current programmes offered to street children are not adequately tailored to address the multifaceted and interrelated social systems of street children.
11.	Education and Care: How Teachers Promote the Inclusion of Children and Youth at Risk in South Africa	Balie, L. & Sayed, Y. (2020).	Qualitative: Phenomenological approach.	Participants included the centre manager, an educational psychologist, occupational therapist, inclusive education specialist, and ten teachers. All participants, except for the inclusive education specialist, are employed at a child and youth care centre (CYCC) in the Western Cape. There were 14 participants in total.	Study findings indicated that the *Curriculum of Care* is a better response than the rigid national curriculum. It is considered better because the CAPS curriculum inadequately responds to the emotional and psychological barriers that children and youth at risk face with regards to education. Furthermore, teachers can implement the Curriculum of Care in various ways to ensure that youth and children at risk are included in the learning process. Furthermore, most implementations entail building a secure and sustained relationship with the child. The Curriculum of Care entails a holistic approach to education, so the schedule can be full and extensive. Learners would spend half of the day involved in educational activities and the other half in therapeutic and extracurricular activities. The drawback is that a deep learning experience is not always possible.	Children and youth at risk need a curriculum that addresses both affective and cognitive development, ensuring that students are appropriately prepared for life beyond the institution. Furthermore, teachers who implement this curriculum must ensure that behavioural and emotional needs are balanced with academic needs and that all requirements are tended to.
12.	Building Resilience Among Orphaned and Vulnerable Children Through the Memory Book Intervention	Braband, B. J., Faris. T. & Wilson-Anderson, K. (2018).	Qualitative: Narrative inquiry.	A total of 66 OVC and five caregivers; two caregivers from South African homes, three homes in India, and one in Kenya.	The Memory Book intervention primarily focuses on improving resilience among OVC. The study identified themes from caregivers and children pertaining to identity, relationship, coping, hope, and emotion. The themes pertaining to identity and relationships were deemed especially important because they illustrate how children value achievement, a sense of worth, and new relationships. These aspects are important in that, to fill the void left by grief, the development of feelings of love, connectedness and self-esteem are crucial. However, it was found that children placed greater emphasis on identity than relationships. In contrast, the study themes pertaining to coping, hope, and emotion were less prominent. Even so, the themes illustrated how the Memory Book intervention allows children to process repressed feelings towards the loss of their parent/s.	The Memory Book intervention enabled children to acknowledge their emotions and enabled the process of healing. The intervention’s storytelling and drawing components allow these children to develop resilience. Developing resilience is crucial as it allows for healing, the development of self-esteem, and the enhancement of personal and cultural protective factors. By allowing children to develop their identities and relationships with others, the children can experience more hope, agency, and personal control.
13.	Perceptions of Parents/Guardians About the Effectiveness of Future Families Orphans and Vulnerable Children Programme in Olievenhoutbosch, South Africa	Eale, K. E. (2018).	Qualitative: Explorative descriptive design.	Thirteen parents/guardians from the Future Families Programme situated in Olievenhoutbosch.	The study produced three themes pertaining to the effectiveness of the Future Families Programme: (1) service delivery and support mechanisms, (2) perception towards Future Families activities, and (3) attitude towards Future Families activities and staff. Regarding Theme 1, participants appeared to be happy with the services provided by the programme. These services included home visits, educational support, health and nutrition support, psychosocial and social education support, child protection support, parent and guardian support initiatives, and household economic strengthening support. Theme 2 focussed on the participants’ perception regarding susceptibility to OVC conditions, the severity of these conditions, the benefits of the programmes’ activities, and barriers to accessing these activities. Participants could detect when children were at risk and would seek help from the Future Families programme. Furthermore, participants were also able to gauge the severity of a child’s condition and its possible repercussions. Participants also perceived the programme to be of great benefit and value to the children with regard to inter- and intrapersonal aspects. In contrast, participants mentioned barriers that hinder children from participating in the programme, including cultural barriers, lack of awareness of the programme, and participant’s responsibilities outside of the programme. Regarding Theme 3, parents/guardians were all happy and thankful for the programme.	The study concluded that the Future Families programme was effective in its purpose and results. Furthermore, parents/guardians could describe the services delivered by the programme, which activities they perceived to be most effective, and the programme benefits. Additionally, parents and guardians were able to perceive the vulnerability of children, the severity of conditions, and the factors that may make a child more susceptible to the development of conditions.
14.	A study of the collaborative process of volunteers in a literacy intervention programme in support of vulnerable children in South Africa	Zoetmulder, A. (2019).	Qualitative: Participatory action research.	Six volunteers involved with the language and literacy programme of the Durban Child and Youth Centre (DCYCC).	Volunteers, in conjunction with language and speech therapists, were involved in providing language and literacy support to vulnerable children. The review findings indicated that the volunteers valued literacy, had positive experiences of volunteering, and had a sense of civic responsibility and empathy towards the children. Furthermore, collaboration among the volunteers and with speech-language therapists was well established due to a strong sense of common cause, vulnerability and trust, a well-structured action plan, self-reflection, and a passion to be agents of change. Additionally, volunteers were able to make changes to the interventions at a volunteer and programme level. Thereby ensuring that the programme was streamlined and appropriate as to ensure that the programme enriches the literacy of children.	Volunteers showed strong emotional and relational ties with the children in the programme, which strengthened the volunteers’ commitment and passion to the programme. Furthermore, the relational ties among volunteers also strengthened their commitment and passion to the programme. Collaboration was further evident in the action plan execution and changes that were made at a programme level. As a result of this, volunteers had a greater sense of purpose with regard to the programme.
15.	Investigating the effectiveness of orphans and vulnerable children (OVC) programmes in schools: a case of Ntuzuma G-section in Durban	Mbatha, Z. P. (2014).	Qualitative: social constructivism.	Fourteen participants consisting of principles, co-ordinators, educators, caregivers and district co-ordinators from three schools within the Ntuzuma G-section KwaZulu-Natal.	Findings of the study indicated that school principals had sufficient training and experience in executing school-based OVC programmes. However, teachers indicated that they did not have sufficient training especially when facing psychological concerns of the children. Regardless, all participants indicated that they were committed to making a difference in the lives of OVC. With regard to the sustainability of the programme, principles indicated that the number of OVC exceeded that of the resources made available from the government. This not only put the sustainability of the programme under question but also hindered multi-sectoral collaboration. All principles showed concern with regard to the psychological well-being of the children since they are orphaned and or vulnerable. Another point of contrition is that of the difficulty community members faced when recruiting OVC into the programme. Additionally, district officials were not involved enough, according to the participants, as they did not visit the schools and relied on the credibility of the OVC coordinators.	The study concluded that, although schools had improved their abilities to address the needs of OVC, resource constraints jeopardised the sustainability of these improvements. It is, therefore, important to remember that it is not only schools that can support OVC. All participants indicated that they were grateful for the interventions implemented with regard to the educational, psychological, nutritional, and emotional needs of the OVC. However, the interventions in place were not sufficient to address the needs of OVC.
16.	A formative evaluation of the James House programme for orphans and vulnerable children	Mutenheri, H. (2014).	Descriptive research with quantitative approach.	Participants consisted of households currently in the programme who completed the programme and re-entered the programme (*n* *=* *11, 7* and *3*, respectively). Child youth care workers (*n* *=* *7*) and the programme manager were also involved.	The James House Programme provides access to health care, community-based care, education, food, government grants, psychological support, and protection. The theory behind the James House programme was found to be rational. However, evaluation of the programme indicated a mixture of successes and challenges. The programme's clients could easily access psychological, education, health care, government grants and other recreational services. However, due to resource constraints, the programme could not provide OVC with food parcels. Referral services provided by the programme were also successful. The programme was also able to successfully network with a variety of stakeholders to provide supplementary services.	The services provided by the James House programme appeared to be meeting the needs of its clients. Clients were also satisfied with the services and treatment received. However, the programme did show constraints with regard to training provided to childcare workers and the ability to provide food parcels.
17.	Promoting uptake of child HIV testing: an evaluation of the role of a home visiting program for orphans and vulnerable children in South Africa	Thurman, T. R., Luckett, B., Taylor, T. & Carnay, M. (2016b).	Quasi-experimental: propensity score matching with survey data.	Participants included a total of 763 households. Where 282 were individuals previously enrolled in the Future Family home visit programme, and 481 were newly enrolled individuals.	To promote the uptake of HIV testing in children, support was provided in the form of material support, counselling, and referral to a variety of health and social services. Results of the study indicated that 49% of the orphans who were previously enrolled in the home visit programme were tested for HIV. Whereas only 24% of children who were newly enrolled on the programme were tested. Models produced within the study indicated that younger children were more likely to be tested, with the probability of testing decreasing with 6% per year as age increased. Furthermore, children with male guardians had 68% lower odds of being tested when compared to those with female guardians. Having a guardian who is 25 years of age or younger reduces the child’s odds of being tested by 82%. Children cared for by biological or non-biological caregivers had no differences in the probability of being tested. However, if a biological caregiver had HIV, the odds of testing tripled. Furthermore, if the caregiver had prior knowledge of HIV testing, the child would have a 70% greater probability of being tested. When comparing similar households, children who were enrolled in the programme had a 97% increase in odds of being tested.	The Future Families home visiting programme makes a concerted effort to test children and other beneficiaries for HIV. The programme incorporated HIV testing focussed modules for volunteers, refresher courses, encouragement from staff and community resource information provision. However, despite encouraging results, as many as 51% of orphans within the study had never been tested for HIV. It was also found that point-of-service issues hindered children from being tested for HIV.

**Table A2.** Study demographics.

Author	Design	Data collection	Sources of data
Balie, L. & Sayed, Y. (2020).	Qualitative	Semi-structured interviews and focus groups	Individuals who work with or care for OVC
Braband, B. J., Faris. T. & Wilson-Anderson, K. (2018).	Qualitative	Individual interviews with open-ended questions	Individuals who work with or care for OVC and OVC
Breckenridge, T. A., Black-Hughes, C., Rautenbach, J., & McKinley, M. (2019).	Qualitative	Observational methods	OVC only
Eale, K. E. (2018).	Qualitative	Face-to-face, in-depth, semi-structured interviews	Individuals who work with or care for OVC
Kwatubana, S. & Ebrahim, M. (2020).	Qualitative	Semi-structured interviews and document analysis	Individuals who work with or care for OVC
Marais, L., Sharp, C., Pappin, M., Rani, K., Skinner, D., Lenka, M., Cloete, J. & Serekoane, J. (2013).	Mixed methods	Interviews, measurements, and documentation	Individuals who work with or care for OVC and OVC
Mbatha, Z. P. (2014).	Qualitative	Semi-structured interviews	Individuals who work with or care for OVC
Mokomane, Z. & Makoae, M. (2015).	Qualitative	Semi-structured interviews	Individuals who work with or care for OVC
Mufalali, R. M., Makua, M. G. & Matlhaba, K. L. (2022).	Qualitative	World Cafe	OVC only
Mutenheri, H. (2014).	Quantitative	Questionnaires	Individuals who work with or care for OVC and OVC
Mwoma, T. & Pillay, J. (2015).	Mixed methods	Unstructured interviews and structured questionnaires	Individuals who work with or care for OVC and OVC
Sitienei, E. C. & Pillay, J. (2019).	Qualitative	Individual interviews, focus groups, and autobiographies	OVC only
Thupayagale-Tshweneagae, G. & Mokomane, Z. (2014).	Qualitative	Focus group discussions, reflective diaries, and recording of grades	OVC only
Thurman, T. R., Kidman, R., Carton, T. W. & Chiroro, P. (2016a).	Quantitative	Surveys	OVC only
Thurman, T. R., Luckett, B., Taylor, T. & Carnay, M. (2016b).	Quantitative	Surveys	OVC only
Visser, M., Zungu, N. & Ndala-Magoro, N. (2015).	Mixed methods	Questionnaires and focus groups	OVC only
Zoetmulder, A. (2019).	Qualitative	Focus groups, interviews, and observations	Individuals who work with or care for OVC

**Table A3.** Codes and categories produced using content analysis.

Meaning unit	Code	Category
1. OVC felt that their mental needs were met, which led to positive coping mechanisms (Mufalali et al., 2022).	Psychological support	Psychological interventions
2. OVC received support mentorships and peer-group support (Sitienei & Pillay, 2019).		
3. The ISIBINDI project provided personal guidance and counselling support (Visser et al., 2015).		
4. CHHs received psychological support in the form of counselling sessions (Kwatubana & Ebrahim, 2020).		
5. Community-based organisations provided counselling and psychological support (Marais et al., 2013).		
6. OVC received training and education to help them openly communicate their thoughts, be assertive about their needs and develop problem-solving skills (Thupayagale-Tshweneagae & Mokomane, 2014).		
7. The study employed cognitive behavioural strategies; significant improvements were found with regard to self-expression (Breckenridge et al., 2019).		
8. All shelters provided support in the form of therapeutic programmes (Mokomane & Makoae, 2015).		
9. Participants were grateful for the psychological support provided (Mbatha, 2014).		
10. Individuals who participated in the James House programme were able to get access to support for psychological needs (Mutenheri, 2014).		
11. Children received counselling support to promote the uptake of HIV testing in children (Thurman et al., 2016a).		
1. The ISIBINDI project provided support in the form of provision of aid with regard to education and career guidance (Visser et al., 2015).	Educational support	Educational interventions
2. OVC received motivation and educational support from teachers (Mwoma & Pillay, 2015).		
3. Curriculum of Care is a better alternative to the CAPS system to address the academic barriers faced by at-risk youth in educational settings (Balie & Sayed, 2020).		
4. Participants appeared to be happy with the service delivery and educational support received (Eale, 2018).		
5. Participants were grateful for the support provided with regard to educational aspects (Mbatha, 2014).		
6. Individuals who participated in the James House programme were able to obtain access to support pertaining to their educational needs (Mutenheri, 2014).		
7. OVC received food hampers, school uniforms, and materials, as well as payment of tuition and school excursion fees (Sitienei & Pillay, 2019).		
1. The ISIBINDI project provided access to safe parks and food gardens (Visser et al., 2015). 2. The community-based organisations provided food packages, nutritional supplements, and provision of care facilities such as children’s homes, temporary shelters, and daycare facilities (Marais et al., 2013). 3. Participants appeared to be pleased with the service delivery and support received in the form of nutrition support (Eale, 2018). 4. Participants in the James House programme had access to support that provided protection from exploitation or abuse (Mutenheri, 2014). 5. Material support was provided to promote the uptake of HIV testing in children (Thurman et al., 2016b).	Physical resource support	Physical resource interventions
1. OVC received life-skills training (Sitienei & Pillay, 2019). 2. The ISIBINDI project also provided life skills training (Visser et al., 2015).	Psychoeducational support	Psychoeducational interventions
3. CHHs received HIV/AIDS education (Kwatubana & Ebrahim, 2020) 4. The Vhutshilo intervention focused on behavioural changes by addressing topics such as alcohol and substance abuse, crime and sexual violence, HIV/AIDS, healthy sexual relationships, transactional sex, and condom use (Thurman et al., 2016a). 5. The community-based organisations provided life skills and information education by means of support groups (Marais et al., 2013). 6. OVC received training and education about understanding the concept of death and dying. The inevitable goal was to improve OVC comprehension of the death of their parent/s (Thupayagale-Tshweneagae & Mokomane, 2014).		
1. Interpersonal psychotherapy for groups focused on the provision of emotional support (Thurman et al., 2016a). 2. Significant improvements were noted in the children’s moods (Breckenridge et al., 2019). 3. The Curriculum of Care is a better alternative to the CAPS system with regard to addressing the emotional needs of the children (Balie & Sayed, 2020).	Emotional support	Emotional interventions
4. Participants were grateful for the emotional support provided (Mbatha, 2014). 5. Social workers and pastors from five schools in this particular intervention provided emotional support was to children from CHHs (Kwatubana & Ebrahim, 2020).		
1. The ISIBINDI project provided access to health care and treatment (Visser et al., 2015). 2. The community-based organisations provided social assistance to help enable service access (Marais et al., 2013). 3. Participants in the James House programme was able to obtain access to health care and governmental grants (Mutenheri, 2014). 4. Referral to a variety of health and social services were provided to promote the uptake of HIV testing in children (Thurman et al., 2016b). 5. Participants appeared to be pleased with the service delivery and health care referrals received (Eale, 2018).	Service support	Service interventions
1. The community-based organisations provided prevention and behavioural modification programmes (Marais et al., 2013)	Behavioural support	Behavioural interventions
2. The study employed cognitive behavioural and psychoeducational strategies. Significant improvements were observed with regard to participants playing with other children and behaviours with others (Breckenridge et al., 2019). 3. The Curriculum of Care is a better alternative to the CAPS system regarding behavioural barriers children face in educational settings (Balie & Sayed, 2020).		
1. All shelters provided support in the form of developmental programmes (Mokomane & Makoae, 2015).	Developmental support	Developmental interventions
1. All shelters provided recreational programmes as part of their support services (Mokomane & Makoae, 2015).	Recreational support	Recreational interventions
1. The Memory Book intervention, oriented towards improving resilience, reported an improvement in OVC resilience levels (Braband et al., 2018). 2. OVC were provided with financial support for families (Sitienei & Pillay, 2019). 3. The ISIBINDI project provided home visits and family support (Visser et al., 2015).	Resilience support	Resilience interventions
1. Participants appeared to be pleased with home visits and support provided to parents/guardians as part of the support programme (Eale, 2018).	Family support	Family interventions
1. Volunteers, in conjunction with language and speech therapists, provided language-literacy support to vulnerable children (Zoetmulder, 2019).	Language literacy support	Language literacy interventions
