# Supplementary material for: Interventions currently implemented among orphans in South-Africa: a scoping review
Source: SAHARA J. 2026 Apr 10;23(1):2652841. doi: 10.1080/17290376.2026.2652841 (PMC13072705; doi:10.1080/17290376.2026.2652841)
Supplement: Appendices.docx [file RSAH_A_2652841_SM9565.docx]

**Appendices**

***Tables***

**Table 1**. Data Extraction Table of Eligible Studies

| **No.** | **Title of article** | **Author(s) and date of publication** | **Methodology** | | **Participants** | **Findings or Results** | **Conclusion** |
| --- | --- | --- | --- | --- | --- | --- | --- |
| 1. | Psychosocial Support for Orphaned and Vulnerable Children with HIV/AIDS in Eastern Cape, South Africa. | Mufalali, R. M., Makua, M. G. & Matlhaba, K. L. (2022). | A descriptive qualitative design of appreciative enquiry. | 30 OVC aged between 13 and 17 located in the Maluti sub-village. | | Thanks to the South African Red Cross Society (SARC) Maluti project, the OVC were able to develop positive coping mechanisms due to the provision of incentives, education, indoor and outdoor activities, and gardening skills. These also allowed the children to develop close bonds with fellow peers and caretakers. Children also felt that the intervention addressed their developmental, social, and mental needs. The children also appreciated the small group or individual treatment that was given to them. | The SARC Maluti project has been successful in supporting OVC with regard to their psychosocial needs. Furthermore, OVC can thrive when their basic psychosocial needs are met, which highlights the importance of ensuring that these needs are met. |
| 2. | Psycho-educational and social interventions provided for orphans and vulnerable children at a community-based organisation in Soweto, South Africa. | Sitienei, E. C. & Pillay, J. (2019). | Qualitative: a phenomenological approach. | 12 OVC in Soweto were selected by a community-based organisation focussing on OVC exposed and affected by AIDS. Where five were males and seven were females. | | The study identified psychological, educational, and social interventions to be present. Findings suggest that OVC gain significantly from mentorships and peer-group support regarding psychological support. However, it was also found that some participants (*n = 2*) found it difficult to trust mentors or peers. Educational interventions entailed the provision of educational material, food hampers, payment of tuition fees, and payment of excursions and sports activities. Social interventions entailed the OVC learning life skills and receiving financial support for their families. However, the sustainability of the financial support provided is uncertain. | The study concluded that, although the intervention efforts are successful, there is a need for comprehensive support networks to address psychological and social issues. Additionally, counselling services and income-generating projects ought to be a focus within OVC communities affected by HIV/AIDS. |
| **No.** | **Title of article** | **Author(s) and date of publication** | **Methodology** | **Participants** | | **Findings or Results** | **Conclusion** |
| 3. | ISIBINDI, creating circles of care for orphans and vulnerable children in South Africa: post-programme outcomes. | Visser, M., Zungu, N. & Ndala-Magoro, N. (2015). | Mixed methods: quasi-experimental design with focus group discussions (FGD). | A total of 604 OVC were chosen as participants and were identified from all twelve sites of the ISIBINDI community-based intervention. These sites are situated in Kwazulu-Natal, Eastern Cape, Mpumalanga, and Gauteng. Participants included 427 ex-participants of the intervention and 177 control group members. All participants were 18–25 years old. | | The ISIBINDI project provided support in the following ways: (1) home visits and family support, (2) personal guidance and counselling, (3) access to a safe park, (4) provision of aid with regards to education and career guidance, (5) life skill training, and (6) access to health care and treatment. Quantitative analysis indicated that ex-participants of the intervention had higher mean self-esteem scores when compared to the control group (M = 5.01 and 3.82, respectively; p-value < .01). Ex-participants also had higher mean problem-solving skills when compared to the control group (M = 3.97 and 2.26 respectively; p-value < .05). Family support was also higher in ex-participants when compared to control groups (M = 4.62 and 3.31 respectively, p-value < .05). Ex-participants also had lower HIV-risk behaviours when compared to the control group (M = 12.9 and 19.7 respectively, p-value < .05). Qualitative results indicated overall improvement in psychological well-being; especially pertaining to self-esteem, problem-solving skills, and family communication and relationships. | The evaluation of the ISIBINDI programme found that this intervention, on a multi-faceted level, provided positive outcomes that allowed for protective barriers to OVC. |
| **No.** | **Title of article** | **Author(s) and date of publication** | **Methodology** | **Participants** | | **Findings or Results** | **Conclusion** |
| 4. | Psychosocial Support Provision for Learners from Child-Headed Households in Five Public Schools in South Africa. | Kwatubana, S. & Ebrahim, M. (2020). | Qualitative: a phenomenological approach. | Participants consisted of principles (*n = 5*), teachers (*n = 9*), and health programme coordinators (*n = 5*) from five schools in the Sedibeng East district. Nineteen participants were selected based on who attended to the most Child-Headed Household (CHH) students. | | Results of the study indicated that CHH students received material, emotional, and spiritual support from various sources. With regards to physical and material needs, the National School Nutrition Programme, uniform programme, Sanitary Pads Project, Food Garden Project, Nature’s Choice, and school programmes involved with the provision of adult supervision were present. These programmes and projects aided CHH individuals with food, school uniforms, and adult supervision in various ways. With regards to emotional and psychological support, all schools had social workers who frequently made visits to the school for counselling sessions, in addition to one school that had counsellors and nurses from Lifeline educate students on HIV/AIDS and teenage pregnancy. Furthermore, two of the schools arranged for pastors to visit their schools and provide guidance to students. All programmes, projects, and interventions originated from either governmental or non-governmental organisations. | The study found that although a variety of interventions were present, their efficacy remained uncertain. Furthermore, there is a need for schools to change their health policies and school-wide practices to be more accommodating of interventions that can target CHH students specifically.  Teachers also require further training on trauma-sensitive practices as well as stress, burnout, and secondary trauma when working with children from CHHs. Additionally, more collaboration between teachers and social workers is required to ensure that a multipronged approach can be feasible. |
| **No.** | **Title of article** | **Author(s) and date of publication** | **Methodology** | **Participants** | | **Findings or Results** | **Conclusion** |
| 5. | Psychological and behavioral interventions to reduce HIV risk: evidence from a randomized control trial among orphaned and vulnerable adolescents in South Africa. | Thurman, T. R., Kidman, R., Carton, T. W. & Chiroro, P. (2016). | Quantitative: randomised control trail. | Students aged 14 –17 years old enrolled in the World Vision South Africa community-based Networks of Hope OVC programme. The Networks of Hope programme offers two interventions: (1) interpersonal psychotherapy for groups and (2) a curriculum-based behavioural intervention. Students were in 84 villages in two districts in the Eastern Cape. This equated to 1,016 participants. | | Interpersonal psychotherapy for groups focused on improving interpersonal skills and providing emotional support. Whereas the Vhutshilo intervention focused on behavioural changes by addressing topics such as alcohol and substance abuse, crime and sexual violence, HIV/AIDS, healthy sexual relationships, transactional sex, and condom use. The review found that only significant intervention effects were present when individuals participated in both interventions. Furthermore, effects also varied by gender. For girls, condom use increased by approximately 32% when compared to baseline survey results. Whereas in the control group, only a 7% increase in condom use occurred. With regards to boys, the prevalence of risky sexual behaviour was significantly lower when compared to the control group. Furthermore, the predicted probability of engaging in risky sexual behaviour increased by 9% in boys from the control group. In comparison, the predicted probability of engaging in risky sexual behaviour stayed the same for boys who participated in both interventions. | The study concluded that, although the interventions were effective, they functioned in conjunction with existing interventions that provided aid for economic and educational needs. The study also concluded that community-based interventions that rely on local community members and deliver services directly to OVC and their households are effective and provide a promising model for other interventions. Additionally, the combination of theory-driven psychological and behavioural intervention packages is promising and should be promoted amongst OVC. |
| **No.** | **Title of article** | **Author(s) and date of publication** | **Methodology** | **Participants** | | **Findings or Results** | **Conclusion** |
| 6. | Community-based mental health support for orphans and vulnerable children in South Africa: a triangulation study. | Marais, L., Sharp, C., Pappin, M., Rani, K., Skinner, D., Lenka, M., Cloete, J. & Serekoane, J. (2014). | Mixed methods. | The study consisted of two sets of participants. One set was management and staff members of community-based organisations that serve OVC within the Mangaung Municipality, Free State. The second set consisted of 607 OVC from the same area. | | Findings showed that the community-based organisations had eight aims aligned with those the Free State Provincial Government laid out. These aims: (1) the provision of care services, (2) the provision of support services, (3) attending to psychological needs, (4) behavioural prevention and modification programmes, (5) the provision of training programmes, (6) provision of social assistance, (7) community mobilisation, and (8) provision of care facilities. Qualitative results indicated that all the CBOs in the area had different conceptualisations of their purposes. However, all the CBOs agreed that alleviating poverty was their most important aspect. Quantitative analysis indicated that only three of the aims had a positive statistically significant relationship with the scores obtained on the Strengths and Difficulties Questionnaire (SDQ). Access to medical services, food expenditure and total expenditure were related to positive mental health outcomes. | The study produced four conclusions. First, funding guidelines provided by the government influence how CBOs position themselves regarding their purpose. Second, CBOs are mainly there to help the government carry out what the government considers to be its own work. Third, guidelines make little mention directly of the mental health of OVC and that HIV/AIDS is more a socio-economic problem. Fourth, cash transfers were not an effective means of providing intervention. |
| **No.** | **Title of article** | **Author(s) and date of publication** | **Methodology** | **Participants** | | **Findings or Results** | **Conclusion** |
| 7. | Educational support for orphans and vulnerable children in primary schools: Challenges and interventions | Mwoma, T. & Pillay, J. (2016). | A mixed methods approach involving descriptive and qualitative designs. | Participants were teachers and Grade 7 students from public primary schools in Soweto. A total of 107 individuals participated in the study, with 42 being teachers and 65 being students (43 boys and 22 girls). | | The government provided Interventions to pay OVC’s school fees in addition to providing their books and stationery. Furthermore, governmental soup kitchens provided OVC with meals. External organisations were involved in providing students with school uniforms, while teachers played an active role in motivating and aiding OVC students who struggled academically. Although most of the OVC students corroborated the above-mentioned provisions, there is a small percentage of OVC students not receiving these services. Teaches reported that they do not have enough time to attend to the needs of OVC students, with the time pressure to finish curriculum work with students in addition to working long hours. OVC students displayed many challenges. They were marginalised by peers and other teachers, had reading, and writing difficulties, had low self-esteem, often displayed mood disorders and had difficulties concentrating in class and on schoolwork. Furthermore, OVC students also had greater rates of absenteeism or lateness in school attendance. With regard to meal provision, both students and teachers felt that the needs of the OVC far exceed the available resources. OVC students would often come to school hungry as there is no food at home, which poses another problem in that meal provisions can only be done for students and not their family members. | Although the government has put in place numerous means by which OVC can be aided, there are still OVC needs that are unmet. Teachers require further training with regard to engaging with so many OVC. Food programmes ought to be implemented at the homes of the OVC, and education is needed among parents and guardians pertaining to the importance of school attendance and homework completion by the OVC. |
| **No.** | **Title of article** | **Author(s) and date of publication** | **Methodology** | **Participants** | | **Findings or Results** | **Conclusion** |
| 8. | Evaluation of a peer-based mental health support program for adolescents orphaned by AIDS in South Africa. | Thupayagale-Tshweneagae, G. & Mokomane, Z. (2014). | Qualitative: phenomenological approach with focus group discussions. | Participants were 15 adolescents aged 14 to 18 who were orphaned as a result of AIDS and who participated in the Better Accept Reality (BAR) programme. | | During the evaluation process, it was noted that most of the participants became more conversive with regard to discussing death and dying and coming to terms with accepting the death of their parent or parents. Listening skills and expression of thoughts also improved among most participants. Furthermore, most of the participants also become more assertive about their needs, thoughts, and feelings. Additionally, problem-solving skills also improved for most of the participants. Participants also found that their relationship with their caregivers had improved, although some reported that it was too early for them to respect their caregivers after being treated so poorly by them. The final observation was that some participants improved their school grades; however, this was a small number of participants (*n = 4*). | The study findings indicated that the BAR programme effectively improves participants’ psychological well-being. Furthermore, the reorientation of peer-based mental health programmes for adolescents orphaned as a result of AIDS ought to be considered because psycho-educational components can help improve the mental health of these individuals. Furthermore, these programmes should be implemented at schools. Aspects such as family support and caregiver commitment ought to be taken into account when orphan placement is considered. |
| **No.** | **Title of article** | **Author(s) and date of publication** | **Methodology** | **Participants** | | **Findings or Results** | **Conclusion** |
| 9. | HIV/AIDS orphans in South Africa: NGO interventions supporting transitions to alternative care. | Breckenridge, T. A., Black-Hughes, C., Rautenbach, J., & McKinley, M. (2019). | Qualitative: phenomenological approach. | Forty-nine orphans in a small rural village in the Eastern Cape. | | The NGO interventions employed psychoeducational and cognitive behavioural strategies. The study found themes pertaining to the bereavement displayed by children towards their parent/s death or absence, a need for physical or tactile attention, negative behaviours at the beginning of the intervention and an overall improvement at the end of the intervention. Furthermore, differences were found in children who were orphaned due to different causes (due to HIV/AIDS, unknown or abandonment). With regards to bereavement, most of the children displayed sadness and crying; some were not aware of their parents’ death, some experienced guilt, some refused to talk about their parents’ death, denial, anger, worry, and were haunted by the death of their parent/s, scared, some ran away from their caregivers, some blamed others, shyness and isolation. With regards to touch and affection, six required physical and tactile attention. Of these six children, three required affirmation that they are loved. Furthermore, only eight of the OVC displayed happiness and satisfaction with their caregivers. Psychoeducational support provided to the children, community and caregivers allowed for improvement pertaining to mood and interpersonal relationships and interactions within 35 of the OVC. Furthermore, the children found it easier to play with others, express themselves and showed improvement in behaviours. | The NGO focused on developing healthy lifestyles, providing leadership skills and daily living skills, providing education, and enhancing self-esteem. Destructive and low self-esteem behaviours declined over six months. Additionally, the study showed that OVC requires interventions that tap into their social, educational, and psychological needs. |
| **No.** | **Title of article** | **Author(s) and date of publication** | **Methodology** | **Participants** | | **Findings or Results** | **Conclusion** |
| 10. | An overview of programmes offered by shelters for street children in South Africa | Mokomane, Z. & Makoae, M. (2015). | Qualitative approach with semi-structured interviews. | Social workers, shelter managers, and the provincial and district authorities are responsible for the implementation of the Children’s Act and Norms and Standards. The individuals were from two districts situated in four provinces in South Africa (two per district, per province). | | The study found that all shelters in all the study districts adhered to the Children’s Act and Norms and Standards. Furthermore, all the shelters provided interventions in the form of developmental, recreational and therapeutic programmes. | The study concluded that current national legislative and policy frameworks in South Africa align with the rights and protection afforded to street children internationally. Furthermore, the programme focuses on early intervention and can be deemed an appropriate means of providing aid to children. A point of contrition, however, is that all shelters predominantly focus on psychosocial support and life skill training as a means of altering children’s behaviours. There is also a widespread emphasis on enrolling street children into local mainstream schools that cannot attend to their needs pertaining to developmental delays. Programmes offered by shelters are also centred around children and exclude other key stakeholders. As a final point, despite the comprehensive legislative and policy frameworks, the current programmes offered to street children are not adequately tailored to address the multifaceted and interrelated social systems of street children. |
| **No.** | **Title of article** | **Author(s) and date of publication** | **Methodology** | **Participants** | | **Findings or Results** | **Conclusion** |
| 11. | Education and Care: How Teachers Promote the Inclusion of Children and Youth at Risk in South Africa | Balie, L. & Sayed, Y. (2020). | Qualitative: Phenomenological approach. | Participants included the centre manager, an educational psychologist, occupational therapist, inclusive education specialist, and ten teachers. All participants, except for the inclusive education specialist, are employed at a child and youth care centre (CYCC) in the Western Cape. There were 14 participants in total. | | Study findings indicated that the *Curriculum of Care* is a better response than the rigid national curriculum. It is considered better because the CAPS curriculum inadequately responds to the emotional and psychological barriers that children and youth at risk face with regards to education. Furthermore, teachers can implement the Curriculum of Care in various ways to ensure that youth and children at risk are included in the learning process. Furthermore, most implementations entail building a secure and sustained relationship with the child. The Curriculum of Care entails a holistic approach to education, so the schedule can be full and extensive. Learners would spend half of the day involved in educational activities and the other half in therapeutic and extracurricular activities. The drawback is that a deep learning experience is not always possible. | Children and youth at risk need a curriculum that addresses both affective and cognitive development, ensuring that students are appropriately prepared for life beyond the institution. Furthermore, teachers who implement this curriculum must ensure that behavioural and emotional needs are balanced with academic needs and that all requirements are tended to. |
| **No.** | **Title of article** | **Author(s) and date of publication** | **Methodology** | **Participants** | | **Findings or Results** | **Conclusion** |
| 12. | Building Resilience Among Orphaned and Vulnerable Children Through the Memory Book Intervention | Braband, B. J., Faris. T. & Wilson-Anderson, K. (2018). | Qualitative: Narrative inquiry. | A total of 66 OVC and five caregivers; two caregivers from South African homes, three homes in India, and one in Kenya. | | The Memory Book intervention primarily focuses on improving resilience among OVC. The study identified themes from caregivers and children pertaining to identity, relationship, coping, hope, and emotion. The themes pertaining to identity and relationships were deemed especially important because they illustrate how children value achievement, a sense of worth, and new relationships. These aspects are important in that, to fill the void left by grief, the development of feelings of love, connectedness and self-esteem are crucial. However, it was found that children placed greater emphasis on identity than relationships. In contrast, the study themes pertaining to coping, hope, and emotion were less prominent. Even so, the themes illustrated how the Memory Book intervention allows children to process repressed feelings towards the loss of their parent/s. | The Memory Book intervention enabled children to acknowledge their emotions and enabled the process of healing. The intervention’s storytelling and drawing components allow these children to develop resilience. Developing resilience is crucial as it allows for healing, the development of self-esteem, and the enhancement of personal and cultural protective factors. By allowing children to develop their identities and relationships with others, the children can experience more hope, agency, and personal control. |
| **No.** | **Title of article** | **Author(s) and date of publication** | **Methodology** | **Participants** | | **Findings or Results** | **Conclusion** |
| 13. | Perceptions of Parents/Guardians About the Effectiveness of Future Families Orphans and Vulnerable Children Programme in Olievenhoutbosch, South Africa | Eale, K. E. (2018). | Qualitative: Explorative descriptive design. | Thirteen parents/guardians from the Future Families Programme situated in Olievenhoutbosch. | | The study produced three themes pertaining to the effectiveness of the Future Families Programme: (1) service delivery and support mechanisms, (2) perception towards Future Families activities, and (3) attitude towards Future Families activities and staff. Regarding Theme 1, participants appeared to be happy with the services provided by the programme. These services included home visits, educational support, health and nutrition support, psychosocial and social education support, child protection support, parent and guardian support initiatives, and household economic strengthening support. Theme 2 focussed on the participants’ perception regarding susceptibility to OVC conditions, the severity of these conditions, the benefits of the programmes’ activities, and barriers to accessing these activities. Participants could detect when children were at risk and would seek help from the Future Families programme. Furthermore, participants were also able to gauge the severity of a child’s condition and its possible repercussions. Participants also perceived the programme to be of great benefit and value to the children with regard to inter- and intrapersonal aspects. In contrast, participants mentioned barriers that hinder children from participating in the programme, including cultural barriers, lack of awareness of the programme, and participant’s responsibilities outside of the programme. Regarding Theme 3, parents/guardians were all happy and thankful for the programme. | The study concluded that the Future Families programme was effective in its purpose and results. Furthermore, parents/guardians could describe the services delivered by the programme, which activities they perceived to be most effective, and the programme benefits. Additionally, parents and guardians were able to perceive the vulnerability of children, the severity of conditions, and the factors that may make a child more susceptible to the development of conditions. |
| **No.** | **Title of article** | **Author(s) and date of publication** | **Methodology** | **Participants** | | **Findings or Results** | **Conclusion** |
| 14. | A study of the collaborative process of volunteers in a literacy intervention programme in support of vulnerable children in South Africa | Zoetmulder, A. (2019). | Qualitative: Participatory action research. | Six volunteers involved with the language and literacy programme of the Durban Child and Youth Centre (DCYCC). | | Volunteers, in conjunction with language and speech therapists, were involved in providing language and literacy support to vulnerable children. The review findings indicated that the volunteers valued literacy, had positive experiences of volunteering, and had a sense of civic responsibility and empathy towards the children. Furthermore, collaboration among the volunteers and with speech-language therapists was well established due to a strong sense of common cause, vulnerability and trust, a well-structured action plan, self-reflection, and a passion to be agents of change. Additionally, volunteers were able to make changes to the interventions at a volunteer and programme level. Thereby ensuring that the programme was streamlined and appropriate as to ensure that the programme enriches the literacy of children. | Volunteers showed strong emotional and relational ties with the children in the programme, which strengthened the volunteers’ commitment and passion to the programme. Furthermore, the relational ties among volunteers also strengthened their commitment and passion to the programme. Collaboration was further evident in the action plan execution and changes that were made at a programme level. As a result of this, volunteers had a greater sense of purpose with regard to the programme. |
| **No.** | **Title of article** | **Author(s) and date of publication** | **Methodology** | **Participants** | | **Findings or Results** | **Conclusion** |
| 15. | Investigating the effectiveness of orphans and vulnerable children (OVC) programmes in schools: a case of Ntuzuma G-section in Durban | Mbatha, Z. P. (2014). | Qualitative: social constructivism. | Fourteen participants consisting of principles, co-ordinators, educators, caregivers and district co-ordinators from three schools within the Ntuzuma G-section KwaZulu-Natal. | | Findings of the study indicated that school principals had sufficient training and experience in executing school-based OVC programmes. However, teachers indicated that they did not have sufficient training especially when facing psychological concerns of the children. Regardless, all participants indicated that they were committed to making a difference in the lives of OVC. With regard to the sustainability of the programme, principles indicated that the number of OVC exceeded that of the resources made available from the government. This not only put the sustainability of the programme under question but also hindered multi-sectoral collaboration. All principles showed concern with regard to the psychological well-being of the children since they are orphaned and or vulnerable. Another point of contrition is that of the difficulty community members faced when recruiting OVC into the programme. Additionally, district officials were not involved enough, according to the participants, as they did not visit the schools and relied on the credibility of the OVC coordinators. | The study concluded that, although schools had improved their abilities to address the needs of OVC, resource constraints jeopardised the sustainability of these improvements. It is, therefore, important to remember that it is not only schools that can support OVC. All participants indicated that they were grateful for the interventions implemented with regard to the educational, psychological, nutritional, and emotional needs of the OVC. However, the interventions in place were not sufficient to address the needs of OVC. |
| **No.** | **Title of article** | **Author(s) and date of publication** | **Methodology** | **Participants** | | **Findings or Results** | **Conclusion** |
| 16. | A formative evaluation of the James House programme for orphans and vulnerable children | Mutenheri, H. (2014). | Descriptive research with quantitative approach. | Participants consisted of households currently in the programme who completed the programme and re-entered the programme (*n = 11, 7* and *3,* respectively). Child youth care workers (*n = 7*) and the programme manager were also involved. | | The James House Programme provides access to health care, community-based care, education, food, government grants, psychological support, and protection. The theory behind the James House programme was found to be rational. However, evaluation of the programme indicated a mixture of successes and challenges. The programme's clients could easily access psychological, education, health care, government grants and other recreational services. However, due to resource constraints, the programme could not provide OVC with food parcels. Referral services provided by the programme were also successful. The programme was also able to successfully network with a variety of stakeholders to provide supplementary services. | The services provided by the James House programme appeared to be meeting the needs of its clients. Clients were also satisfied with the services and treatment received. However, the programme did show constraints with regard to training provided to childcare workers and the ability to provide food parcels. |
| **No.** | **Title of article** | **Author(s) and date of publication** | **Methodology** | **Participants** | | **Findings or Results** | **Conclusion** |
| 17. | Promoting uptake of child HIV testing: an evaluation of the role of a home visiting program for orphans and vulnerable children in South Africa | Thurman, T. R., Luckett, B., Taylor, T. & Carnay, M. (2016). | Quasi-experimental: propensity score matching with survey data. | Participants included a total of 763 households. Where 282 were individuals previously enrolled in the Future Family home visit programme, and 481 were newly enrolled individuals. | | To promote the uptake of HIV testing in children, support was provided in the form of material support, counselling, and referral to a variety of health and social services. Results of the study indicated that 49% of the orphans who were previously enrolled in the home visit programme were tested for HIV. Whereas only 24% of children who were newly enrolled on the programme were tested. Models produced within the study indicated that younger children were more likely to be tested, with the probability of testing decreasing with 6% per year as age increased. Furthermore, children with male guardians had 68% lower odds of being tested when compared to those with female guardians. Having a guardian who is 25 years of age or younger reduces the child’s odds of being tested by 82%. Children cared for by biological or non-biological caregivers had no differences in the probability of being tested. However, if a biological caregiver had HIV, the odds of testing tripled. Furthermore, if the caregiver had prior knowledge of HIV testing, the child would have a 70% greater probability of being tested. When comparing similar households, children who were enrolled in the programme had a 97% increase in odds of being tested. | The Future Families home visiting programme makes a concerted effort to test children and other beneficiaries for HIV. The programme incorporated HIV testing focussed modules for volunteers, refresher courses, encouragement from staff and community resource information provision. However, despite encouraging results, as many as 51% of orphans within the study had never been tested for HIV. It was also found that point-of-service issues hindered children from being tested for HIV. |

**Table 2**. Study Demographics

| **Author** | **Design** | **Data Collection** | **Sources of Data** |
| --- | --- | --- | --- |
| Balie, L. & Sayed, Y. (2020). | Qualitative | Semi-structured interviews and focus groups | Individuals who work with or care for OVC |
| Braband, B. J., Faris. T. & Wilson-Anderson, K. (2018). | Qualitative | Individual interviews with open-ended questions | Individuals who work with or care for OVC and OVC |
| Breckenridge, T. A., Black-Hughes, C., Rautenbach, J., & McKinley, M. (2019). | Qualitative | Observational methods | OVC only |
| Eale, K. E. (2018). | Qualitative | Face-to-face, in-depth, semi-structured interviews | Individuals who work with or care for OVC |
| Kwatubana, S. & Ebrahim, M. (2020). | Qualitative | Semi-structured interviews and document analysis | Individuals who work with or care for OVC |
| Marais, L., Sharp, C., Pappin, M., Rani, K., Skinner, D., Lenka, M., Cloete, J. & Serekoane, J. (2014). | Mixed methods | Interviews, measurements, and documentation | Individuals who work with or care for OVC and OVC |
| Mbatha, Z. P. (2014). | Qualitative | Semi-structured interviews | Individuals who work with or care for OVC |
| Mokomane, Z. & Makoae, M. (2015). | Qualitative | Semi-structured interviews | Individuals who work with or care for OVC |
| Mufalali, R. M., Makua, M. G. & Matlhaba, K. L. (2022). | Qualitative | World Cafe | OVC only |
| Mutenheri, H. (2014). | Quantitative | Questionnaires | Individuals who work with or care for OVC and OVC |
| Mwoma, T. & Pillay, J. (2016). | Mixed methods | Unstructured interviews and structured questionnaires | Individuals who work with or care for OVC and OVC |
| Sitienei, E. C. & Pillay, J. (2019). | Qualitative | Individual interviews, focus groups, and autobiographies | OVC only |
| Thupayagale-Tshweneagae, G. & Mokomane, Z. (2014). | Qualitative | Focus group discussions, reflective diaries, and recording of grades | OVC only |
| Thurman, T. R., Kidman, R., Carton, T. W. & Chiroro, P. (2016). | Quantitative | Surveys | OVC only |
| Thurman, T. R., Luckett, B., Taylor, T. & Carnay, M. (2016). | Quantitative | Surveys | OVC only |
| **Author** | **Design** | **Data Collection** | **Sources of Data** |
| Visser, M., Zungu, N. & Ndala-Magoro, N. (2015). | Mixed methods | Questionnaires and focus groups | OVC only |
| Zoetmulder, A. (2019). | Qualitative | Focus groups, interviews, and observations | Individuals who work with or care for OVC |

**Table 3**. Codes and Categories Produced using Content Analysis

| **Meaning Unit** | **Code** | **Category** |
| --- | --- | --- |
| 1. OVC felt that their mental needs were met, which led to positive coping mechanisms (Mufalali et al., 2022). | Psychological support | Psychological interventions |
| 1. OVC received support mentorships and peer-group support (Sitienei & Pillay, 2019). |  |  |
| 1. The ISIBINDI project provided personal guidance and counselling support (Visser et al., 2015). |  |  |
| 1. CHHs received psychological support in the form of counselling sessions (Kwatubana & Ebrahim, 2020). |  |  |
| 1. Community-based organisations provided counselling and psychological support (Marais et al., 2014). |  |  |
| 1. OVC received training and education to help them openly communicate their thoughts, be assertive about their needs and develop problem-solving skills (Thupayagale-Tshweneagae & Mokomane, 2014). |  |  |
| 1. The study employed cognitive behavioural strategies; significant improvements were found with regard to self-expression (Breckenridge et al., 2019). |  |  |
| 1. All shelters provided support in the form of therapeutic programmes (Mokomane & Makoae, 2015). |  |  |
| 1. Participants were grateful for the psychological support provided (Mbatha, 2014). |  |  |
| **Meaning Unit** | **Code** | **Category** |
| 1. Individuals who participated in the James House programme were able to get access to support for psychological needs (Mutenheri, 2014). |  |  |
| 1. Children received counselling support to promote the uptake of HIV testing in children (Thurman et al., 2016a). |  |  |
| 1. The ISIBINDI project provided support in the form of provision of aid with regard to education and career guidance (Visser et al., 2015). | Educational support | Educational interventions |
| 1. OVC received motivation and educational support from teachers (Mwoma & Pillay, 2016). |  |  |
| 1. Curriculum of Care is a better alternative to the CAPS system to address the academic barriers faced by at-risk youth in educational settings (Balie & Sayed, 2020). |  |  |
| 1. Participants appeared to be happy with the service delivery and educational support received (Eale, 2018). |  |  |
| 1. Participants were grateful for the support provided with regard to educational aspects (Mbatha, 2014). |  |  |
| 1. Individuals who participated in the James House programme were able to obtain access to support pertaining to their educational needs (Mutenheri, 2014). |  |  |
| **Meaning Unit** | **Code** | **Category** |
| 1. OVC received food hampers, school uniforms, and materials, as well as payment of tuition and school excursion fees (Sitienei & Pillay, 2019). |  |  |
| 1. The ISIBINDI project provided access to safe parks and food gardens (Visser et al., 2015). 2. The community-based organisations provided food packages, nutritional supplements, and provision of care facilities such as children’s homes, temporary shelters, and daycare facilities (Marais et al., 2014). 3. Participants appeared to be pleased with the service delivery and support received in the form of nutrition support (Eale, 2018). 4. Participants in the James House programme had access to support that provided protection from exploitation or abuse (Mutenheri, 2014). 5. Material support was provided to promote the uptake of HIV testing in children (Thurman et al., 2016b). | Physical resource support | Physical resource interventions |
| 1. OVC received life-skills training (Sitienei & Pillay, 2019). 2. The ISIBINDI project also provided life skills training (Visser et al., 2015). | Psychoeducational support | Psychoeducational interventions |
| **Meaning Unit** | **Code** | **Category** |
| 1. CHHs received HIV/AIDS education (Kwatubana & Ebrahim, 2020) 2. The Vhutshilo intervention focused on behavioural changes by addressing topics such as alcohol and substance abuse, crime and sexual violence, HIV/AIDS, healthy sexual relationships, transactional sex, and condom use (Thurman et al., 2016a). 3. The community-based organisations provided life skills and information education by means of support groups (Marais et al., 2014). 4. OVC received training and education about understanding the concept of death and dying. The inevitable goal was to improve OVC comprehension of the death of their parent/s (Thupayagale-Tshweneagae & Mokomane, 2014). |  |  |
| 1. Interpersonal psychotherapy for groups focused on the provision of emotional support (Thurman et al., 2016a). 2. Significant improvements were noted in the children’s moods (Breckenridge et al., 2019). 3. The Curriculum of Care is a better alternative to the CAPS system with regard to addressing the emotional needs of the children (Balie & Sayed, 2020). | Emotional support | Emotional interventions |
| **Meaning Unit** | **Code** | **Category** |
| 1. Participants were grateful for the emotional support provided (Mbatha, 2014). 2. Social workers and pastors from five schools in this particular intervention provided emotional support was to children from CHHs (Kwatubana & Ebrahim, 2020). |  |  |

| 1. The ISIBINDI project provided access to health care and treatment (Visser et al., 2015). 2. The community-based organisations provided social assistance to help enable service access (Marais et al., 2014). 3. Participants in the James House programme was able to obtain access to health care and governmental grants (Mutenheri, 2014). 4. Referral to a variety of health and social services were provided to promote the uptake of HIV testing in children (Thurman et al., 2016b). 5. Participants appeared to be pleased with the service delivery and health care referrals received (Eale, 2018). | Service support | Service interventions |
| --- | --- | --- |
| 1. The community-based organisations provided prevention and behavioural modification programmes (Marais et al., 2014) | Behavioural support | Behavioural interventions |
| **Meaning Unit** | **Code** | **Category** |
| 1. The study employed cognitive behavioural and psychoeducational strategies. Significant improvements were observed with regard to participants playing with other children and behaviours with others (Breckenridge et al., 2019). 2. The Curriculum of Care is a better alternative to the CAPS system regarding behavioural barriers children face in educational settings (Balie & Sayed, 2020). |  |  |
| 1. All shelters provided support in the form of developmental programmes (Mokomane & Makoae, 2015). | Developmental support | Developmental interventions |
| 1. All shelters provided recreational programmes as part of their support services (Mokomane & Makoae, 2015). | Recreational support | Recreational interventions |
| 1. The Memory Book intervention, oriented towards improving resilience, reported an improvement in OVC resilience levels (Braband et al., 2018). 2. OVC were provided with financial support for families (Sitienei & Pillay, 2019). 3. The ISIBINDI project provided home visits and family support (Visser et al., 2015). | Resilience support | Resilience interventions |
| **Meaning Unit** | **Code** | **Category** |
| 1. Participants appeared to be pleased with home visits and support provided to parents/guardians as part of the support programme (Eale, 2018). | Family support | Family interventions |
| 1. Volunteers, in conjunction with language and speech therapists, provided language-literacy support to vulnerable children (Zoetmulder, 2019). | Language literacy support | Language literacy interventions |

***Figures***

**Figure 1**. Literature Selection Process
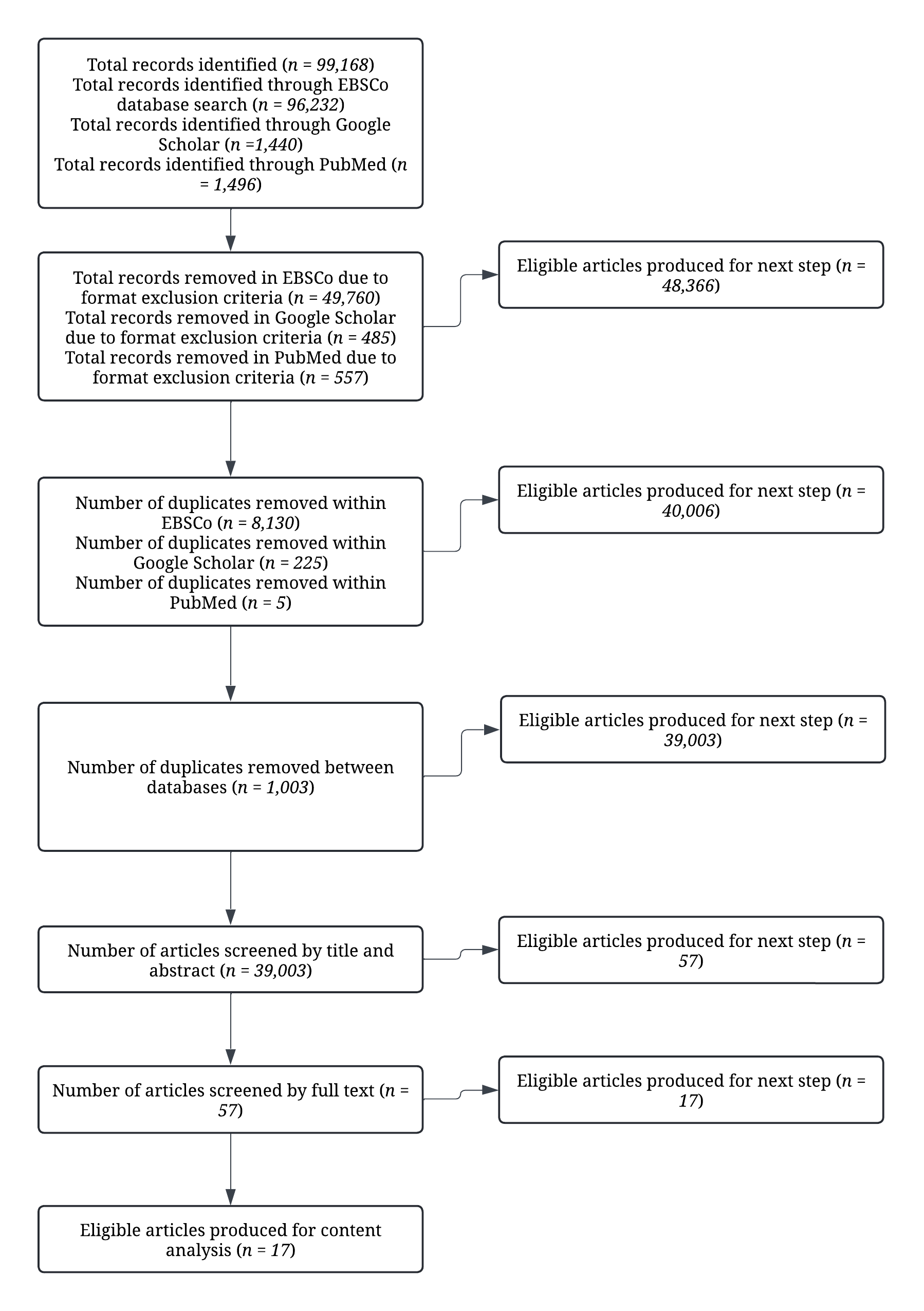


**Figure 2.** Coding Rules and Categorisation Thereof**
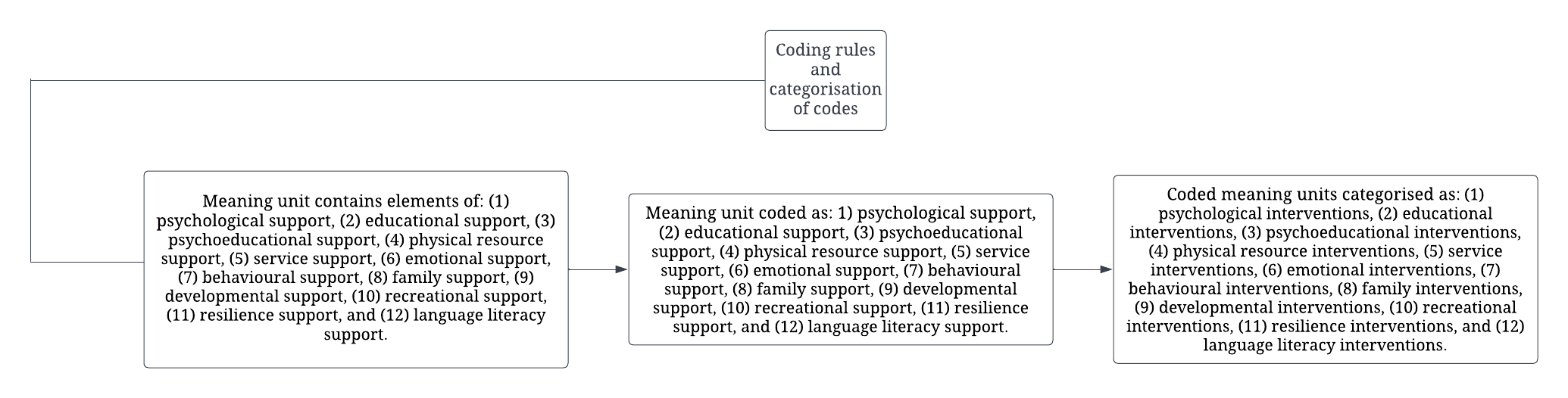
**

*Note.* This figure illustrates that meaning units were coded and categorised according to their respective numbers provided in the figure

**Figure Captions**

1. **Figure 1**. Literature Selection Process
2. **Figure 2.** Coding Rules and Categorisation Thereof
